# Systematic Review and Meta-Analysis of the Efficacy of Interventions Applied during Primary Processing to Reduce Microbial Contamination on Pig Carcasses

**DOI:** 10.3390/foods11142110

**Published:** 2022-07-15

**Authors:** Nevijo Zdolec, Aurelia Kotsiri, Kurt Houf, Avelino Alvarez-Ordóñez, Bojan Blagojevic, Nedjeljko Karabasil, Morgane Salines, Dragan Antic

**Affiliations:** 1 Faculty of Veterinary Medicine, University of Zagreb, 10000 Zagreb, Croatia; nzdolec@vef.hr; 2 Institute of Infection, Veterinary and Ecological Sciences, University of Liverpool, Liverpool L69 3BX, UK; aurelia.kotsiri@liverpool.ac.uk; 3 Faculty of Veterinary Medicine, Department of Veterinary and Biosciences, Ghent University, 9820 Merelbeke, Belgium; kurt.houf@ugent.be; 4 Institute of Food Science and Technology, Department of Food Hygiene and Technology, Universidad de León, 24004 León, Spain; aalvo@unileon.es; 5 Faculty of Agriculture, Department of Veterinary Medicine, University of Novi Sad, 21000 Novi Sad, Serbia; blagojevic.bojan@yahoo.com; 6 Faculty of Veterinary Medicine, University of Belgrade, Department of Food Hygiene and Technology, 11000 Belgrade, Serbia; nedjakn@gmail.com; 7 French Ministry of Agriculture, Office for Slaughterhouses and Cutting Plants, 75015 Paris, France; morgane.salines@agriculture.gouv.fr

**Keywords:** interventions, pig carcasses, aerobic colony count, *Enterobacteriaceae*, generic *E. coli*, *Yersinia*, abattoir, hot water washing, chilling

## Abstract

Interventions from lairage to the chilling stage of the pig slaughter process are important to reduce microbial contamination of carcasses. The aim of this systematic review and meta-analysis was to assess the effectiveness of abattoir interventions in reducing aerobic colony count (ACC), *Enterobacteriaceae*, generic *Escherichia coli*, and *Yersinia* spp. on pig carcasses. The database searches spanned a 30 year period from 1990 to 2021. Following a structured, predefined protocol, 22 articles, which were judged as having a low risk of bias, were used for detailed data extraction and meta-analysis. The meta-analysis included data on lairage interventions for live pigs, standard processing procedures for pig carcasses, prechilling interventions, multiple carcass interventions, and carcass chilling. Risk ratios (RRs) for prevalence studies and mean log differences (MDs) for concentration outcomes were calculated using random effects models. The meta-analysis found that scalding under commercial abattoir conditions effectively reduced the prevalence of *Enterobacteriaceae* (RR: 0.05, 95% CI: 0.02 to 0.12, *I*^2^ = 87%) and ACC (MD: −2.84, 95% CI: −3.50 to −2.18, *I*^2^ = 99%) on pig carcasses. Similarly, significant reductions of these two groups of bacteria on carcasses were also found after singeing (RR: 0.25, 95% CI: 0.14 to 0.44, *I*^2^ = 90% and MD: −1.95, 95% CI: −2.40 to −1.50, *I*^2^ = 96%, respectively). Rectum sealing effectively reduces the prevalence of *Y. enterocolitica* on pig carcasses (RR: 0.60, 95% CI: 0.41 to 0.89, *I*^2^ = 0%). Under commercial abattoir conditions, hot water washing significantly reduced ACC (MD: −1.32, 95% CI: −1.93 to −0.71, *I*^2^ = 93%) and generic *E. coli* counts (MD: −1.23, 95% CI: −1.89 to −0.57, *I*^2^ = 61%) on pig carcasses. Conventional dry chilling reduced *Enterobacteriaceae* prevalence on pig carcasses (RR: 0.32, 95% CI: 0.21 to 0.48, *I*^2^ = 81%). Multiple carcass interventions significantly reduced *Enterobacteriaceae* prevalence (RR: 0.11, 95% CI: 0.05 to 0.23, *I*^2^ = 94%) and ACC on carcasses (MD: −2.85, 95% CI: −3.33 to −2.37, *I*^2^ = 97%). The results clearly show that standard processing procedures of scalding and singeing and the hazard-based intervention of hot water washing are effective in reducing indicator bacteria on pig carcasses. The prevalence of *Y. enterocolitica* on pig carcasses was effectively reduced by the standard procedure of rectum sealing; nevertheless, this was the only intervention for *Yersinia* investigated under commercial conditions. High heterogeneity among studies and trials investigating interventions and overall lack of large, controlled trials conducted under commercial conditions suggest that more in-depth research is needed.

## 1. Introduction

Microbial contamination of pig carcasses (i.e., skin and meat) can arise from numerous sources and operations in abattoirs, from lairage to chilling. The level of contamination depends on the management of animal purchase, lairage conditions and slaughter technologies, which can vary significantly among abattoirs [1,2,3]. The level of hygiene during processing at slaughter and dressing is assessed based on process hygiene criteria (PHC), which includes testing for *Salmonella* presence, aerobic colony count (ACC) and *Enterobacteriaceae* count (EBC) on carcass surfaces before chilling [4]. Microbiological criteria are usually revised according to the current epidemiological status of animal production and new scientific knowledge. For example, the criteria for *Salmonella* proposed in European Food Safety Authority (EFSA) opinions on modernisation of meat inspection in pigs [5] are stricter and allow for only 6% *Salmonella*-positive pig carcasses in one sampling period of 10 weeks in order for an abattoir process to be considered as satisfactory [6]. On the other hand, PHC for *Yersinia enterocolitica* have not been envisaged in the legislation, although pigs are a common source of pathogenic strains causing yersiniosis in humans [7], and this is one of the priority hazards in pork [8]. *Campylobacter* spp., and particularly *C. coli*, is a frequent contaminant of prechilled pig carcass surfaces; however, given its sensitivity to drying and freezing when conventional dry or blast chilling is used, there is a significant decline of this pathogen on pig carcasses post-chilling [5]. Consequently, pig carcasses and pork are not considered an important source of *Campylobacter* in public health context, and it is not a priority hazard for control at the abattoir stage [5]. Common groups of indicator microorganisms, such as ACC, EBC, generic *Escherichia coli* count and total coliforms, are ideal for assessing the hygiene status of pig carcasses due to the fact of their existing higher levels and more uniform distribution on carcass surfaces compared to pathogens [9,10]. Indeed, the overall hygiene performance of pig abattoirs can be assessed by monitoring the ACC, EBC and generic *Escherichia coli* count before and after each specific slaughter operation. Many studies have shown that standard processing procedures, such as scalding, singeing or rectum sealing, reduce the number of indicator bacteria or the presence of pathogens, while dehairing, polishing and carcass splitting increase bacterial contamination [11,12,13,14,15].

Various interventions, usually hazard-based or good hygienic practice (GHP)-based in nature, are used in pig abattoirs to eliminate or reduce pathogens and spoilage bacteria from carcasses. GHP-based measures are prerequisites used at the preslaughter stage (e.g., lairage holding time and feed withdrawal) and during slaughter and carcass dressing (e.g., scalding, singeing, rectum sealing, head removal, knife trimming, carcass washing). More specific, hazard-based interventions, such as various thermal treatments for carcasses (hot water washing, steam pasteurisation), can be used in the prechilling phase, and do not require specific regulatory approval. On the other hand, chemical washes with organic acids and other chemicals undergo stringent risk assessment processes and regulatory approval [1,16]. Finally, carcass dry air chilling (conventional and blast) has some antimicrobial effect that is based on surface drying and can be complemented or replaced with spray chilling (with water or water plus organic acids or other approved chemicals) to increase the antimicrobial effect. However, the specific interventions used vary from country to country and are influenced by the regulatory framework, economic feasibility, seasonal variations, environmental impact, technical constraints and occupational health and safety [1,16].

Numerous studies using different experimental designs have been conducted over the last couple of decades with the aim of investigating the effectiveness of various interventions for pig carcasses. They usually produce different supporting evidence, depending on many factors (sample size, various study conditions, study design, etc.). One way to address the high heterogeneity between different study designs is to conduct a systematic literature review coupled with meta-analysis. This structured process enables the effectiveness of interventions to be measured with reduced bias and increased transparency and can be used to explain the differences in intervention effectiveness between different studies [17]. There is, however, a lack of meta-analysis studies on pig interventions during primary processing. Two meta-analysis studies, which investigated the effects of abattoir interventions and chilling on *Salmonella* only, found significant effects of organic acid washes, hot water washes, steam pasteurisation and chilling in reducing *Salmonella* on pig carcasses [18,19]. However, there are no meta-analysis studies to investigate interventions’ effects in reducing indicator bacteria counts and *Yersinia* spp. on pig carcasses. Therefore, the aim of this study was to conduct a systematic review and meta-analysis of literature data reporting on the effectiveness of a range of interventions applied to pig carcasses during primary processing in abattoirs, on indicator bacteria (i.e., ACC, EBC and generic *Escherichia coli* count) and *Yersinia* spp.

## 2. Materials and Methods

### 2.1. Review Protocol and Research Question

A systematic review of the literature on the contribution of pig abattoir interventions to the reduction of bacterial load on pig carcasses was conducted, with a focus on the pre and post-slaughter production processes in abattoirs, up to and including primary chilling. The review considered evidence on pig interventions’ efficacy available in the public domain, but only primary research studies were used for data extraction and reporting. The review question was: “What is the efficacy of all possible interventions to control microbial contamination on pig carcasses at any stage in the pork production chain from pigs received in the abattoir to the pig carcass chilling inclusive?” The review followed a structured, predefined protocol and PICO framework. The population studied was pigs produced for meat consumption, including their carcasses at primary processing. Relevant outcome measures for interventions were the effectiveness of each intervention in reducing log levels of indicator bacteria (aerobic colony count (ACC), *Enterobacteriaceae* count (EBC) and generic *E. coli* count) and log levels or prevalence of the foodborne pathogens *Salmonella* spp. and *Yersinia enterocolitica/pseudotuberculosis*. Subsequently, it was agreed to exclude data on *Salmonella* from further analysis, as it was found that an insufficient number of studies had been published since the previous systematic review by Young et al. [18] to justify data analysis. Any GHP- and hazard-based interventions applied from the stage of pigs being received in the abattoir lairage up to (and inclusive of) primary chilling in abattoirs were considered relevant.

### 2.2. Review Team and Search Strategy

Relevance screening, relevance confirmation, risk-of-bias assessment and data extraction were conducted by two review team members, and discrepancies were resolved through discussion or by judgment of a third reviewer. All developed protocols are provided in the Supplementary Material. A comprehensive search algorithm was developed and used for the search of peer-reviewed literature. The algorithm was developed by extracting key words from a selection of twenty known relevant primary research articles on pig interventions (different articles per intervention category), and by reviewing and adapting search strategies and key terms of previously published reviews and risk assessments on this and similar topics. Three databases were searched, Scopus, CAB Direct and SciELO. Key terms were combined using the Boolean operator “OR” into three categories: microorganism/outcome (*E. coli*, *Yersinia*, *Salmonella*, *Enterobacteriaceae*, aerobic colony count), intervention (intervention terms) and population (pig terms). The categories were combined using the “AND” operator. The algorithms were pretested using a list of twenty relevant articles (provided in the Supplementary Materials) in Scopus and CAB direct to ensure they could be sufficiently identified. The searched articles spanned a period of 30 years (1990–2021, except SciELO, which encompassed 2002–2021), with no language restrictions imposed. Search verification included reviewing the reference lists of ten relevant review and ten primary research articles (provided in the Supplementary Materials).

### 2.3. Relevance Screening and Eligibility Criteria for Prioritisation

All retrieved citations were first uploaded in Endnote X9.2 and duplicates removed. Remaining citations were then imported into the web-based systematic review platform Rayyan for subsequent relevance screening at the title and abstract level [20]. Each article was screened through its title and abstract using a prespecified relevance screening form, and then its relevance further confirmed after the full article was procured and using the prespecified checklist (see Supplementary Materials). All experimental and observational study designs were considered for data extraction (controlled, challenge and before-and-after trials, and cohort studies). These included studies measuring interventions’ efficacy through the measurement of concentration (such as colony forming units, (CFU)/sample) and/or prevalence (absence or presence) of microorganisms. Intervention application settings were described as commercial (large or small) abattoirs and pilot plants (where industrial equipment was used in nonindustrial settings) as well as research conducted under laboratory conditions. “In vitro” studies (model broth system experiments) were excluded. The interventions were analysed and presented according to five intervention categories: (i) preslaughter, lairage interventions for live pigs; (ii) standard processing procedures for carcasses; (iii) pig carcass prechilling interventions; (iv) carcass chilling; (v) multiple interventions.

### 2.4. Risk of Bias Assessment and Data Extraction

The risk-of-bias (RoB) assessment was conducted for 25 primary research articles. It was performed using a prespecified tool that was adapted to suit the needs of the topic and study designs, from the Cochrane Collaboration’s recommended tools for randomised and non-randomised study designs [21,22]. Two reviewers conducted RoB assessment independently and any disagreements between them were resolved by a third reviewer. The tool was structured into five domains through which bias might be introduced into the results: (1) bias arising from the randomisation process; (2) bias due to the presence of deviations from intended interventions; (3) bias due to the fact of missing outcome data; (4) bias in measurement of the outcome; (5) bias in selection of the reported result. The possible risk-of-bias judgements were: (1) low risk of bias; (2) some concerns; (3) high risk of bias.

Only articles assessed to be at low risk of bias were considered for detailed data extraction. The data extraction tool included targeted questions about intervention (category, specific intervention and detailed description about intervention parameters), population (i.e., live animal, skin and carcass surface), outcomes (microorganisms) measured, comparison group(s) and intervention efficacy results (concentration and prevalence data). Data were first stratified by study design and conditions, then into specific predefined intervention categories and, finally, by different outcome measures (*Yersinia*, ACC, EBC, generic *Escherichia coli* count). Where data in articles were presented only in visual form, such as graphs, and no other extractable data were present in the text, data on microbial reduction were not considered due to the reduced precision, and these articles were excluded. The detailed protocol followed for RoB assessment and data extraction is provided in the Supplementary Materials.

### 2.5. Random-Effect Meta-Analysis and Reporting

Data were first stratified by the study design and conditions (commercial abattoir or laboratory), then into specific groups for interventions and, finally, grouped together for different microbiological outcomes. If comparison groups had three or more trials that were eligible for meta-analysis, then the mean CFU/cm^2^, CFU/100 cm^2^, and their respective standard deviations (SDs) or standard error of means (SEMs) were extracted from studies measuring concentration outcomes. For prevalence outcomes, only the number of positives in each group was extracted. If only the SEM was available, then a pooled SD was calculated. Trials without a direct comparison group were presented in a tabulated form. Random effects models were calculated using R (version 1.3.1093), including packages meta and metaphor [23,24,25]. These were pooled risk ratios (RRs) for prevalence outcomes and pooled log mean differences for concentration outcomes. If the RR was less than 1, this indicated a lower risk in the intervention group compared to the control one, whereas if the RR was greater than 1, it indicated an increased risk for the intervention group, suggesting the intervention may not be effective. Confidence intervals were also extracted. Weights in the random-effects meta-analysis were based on the size of each study (i.e., number of observations). Forest plots were created to summarise the effects and visualise heterogeneity measures. The results were then summarised and presented in a tabulated form with selected forest plots presented in the main text, while the remaining are available in the Supplementary Materials. Heterogeneity was assessed using *I*^2^, which measures the percentage of variability in the effect size, which is not result of sampling error [26,27]. If *I*^2^ values were greater than 50%, heterogeneity was considered as high, values between 25 and 50% were considered as moderate heterogeneity, whereas values less than 25% represented low levels of heterogeneity. A test for heterogeneity was performed (Cochran’s Q-Statistic), which evaluates the null hypothesis that all studies evaluate the same effect. The resultant *p*-values were also presented; values less than 0.05 indicated that the studies were significantly heterogeneous. Therefore, the resultant forest plots can be split into three groups: those that were homogenous (*p* > 0.05 on the test for heterogeneity), those that were moderately heterogeneous (*p* < 0.05, *I*^2^ ≤ 60%) and those that were highly heterogeneous (*p* < 0.05, *I*^2^ > 60%).

## 3. Results

### 3.1. Study Characteristics and Risk of Bias Assessment

**Figure 1:**
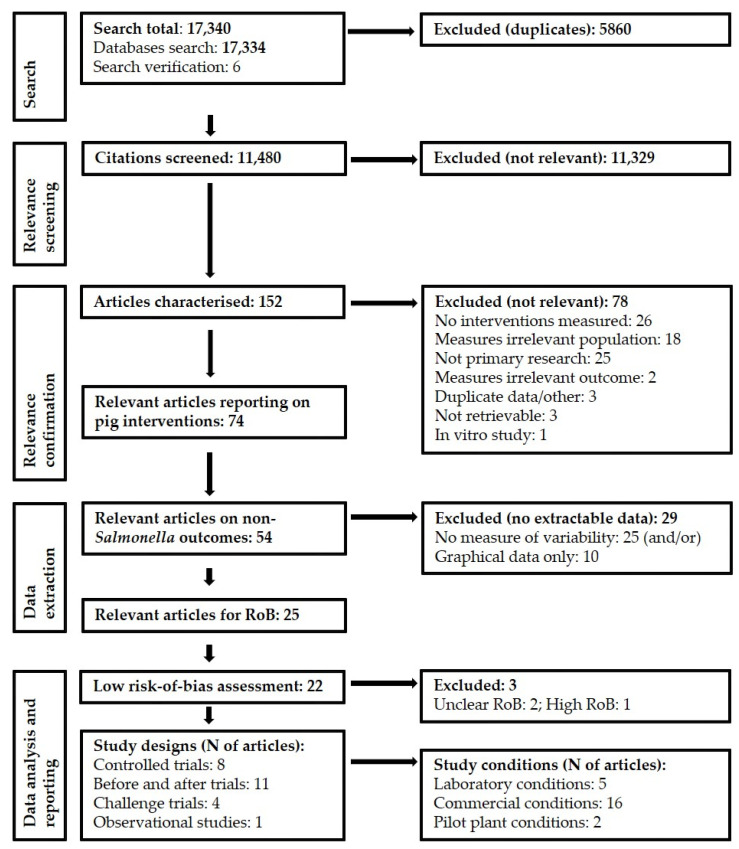
Flow chart of the systematic review and meta-analysis process.

The results from the systematic review, risk-of-bias assessment and data analysis are shown in Figure 1. Of the 17,340 articles retrieved in the database search and search verification, following the deduplication, 11,480 were screened at title and abstract levels for relevance. After screening, 152 articles were procured as full articles and checked for relevance using eligibility criteria, of which 74 reported interventions in pigs from lairage to chilling. For the purpose of this paper, articles reporting data on non-*Salmonella* outcomes (54 in total) were further checked for extractable data (i.e., data with measures of variability and excluding graphical format). The finalised list for subsequent risk-of-bias assessment included 25 articles (key characteristics shown in Table 1). These were twelve before-and-after trials, nine controlled trials, seven challenge trials and one cohort study.

Most studies on interventions in pigs, and the selected outcomes, were conducted in Europe (64%), followed by North America (24%). The majority of studies were conducted under commercial abattoir conditions (69.2%), followed by laboratory conditions (23.1%). Most of the studies investigated pig carcass prechilling interventions, chilling (air, spray and blast chilling) or standard processing procedures/GHP. Scalding and singeing were investigated in four studies each (10.3%) and lairage interventions were investigated in only two studies (5.1%). Among microorganisms, indicator bacteria (predominantly ACC) were investigated the most, and *Yersinia enterocolitica* in only six studies (13.3%) (Table 1).

**Figure 2:**
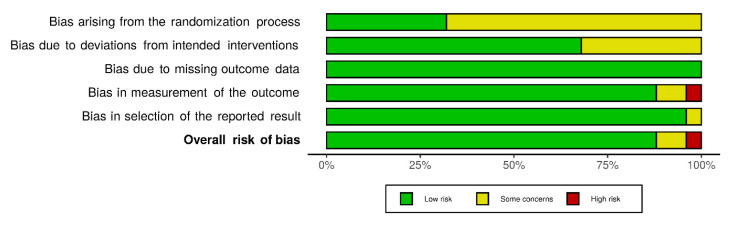
Distribution of risk of bias judgement within each bias domain for all 25 articles investigating pig interventions.

Overall, 22 articles were judged to be at low risk of bias (and progressed to data extraction), two articles had some concerns, and one article was judged to be at high risk of bias. The main concerns for controlled trials, cohort trials and challenge trials were bias arising from the randomisation process, whereas only a limited number of before-and-after trials were associated with a similar risk of bias. The results from the RoB assessment process for the 25 articles are presented in Figure 2 in the form of weighted bar plots of the distribution of risk-of-bias judgements within each bias domain.

### 3.2. Random-Effects Meta-Analysis

**Figure 3:**
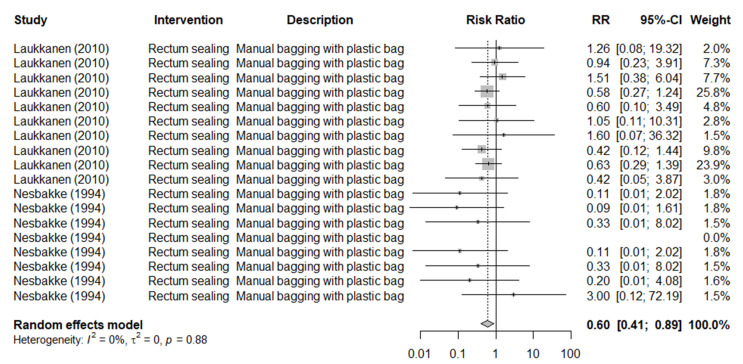
Forest plot of the results of controlled trials performed under commercial abattoir conditions to investigate the efficacy of rectum sealing in reducing *Yersinia enterocolitica* prevalence on pig carcasses [12,34].

**Figure 4:**
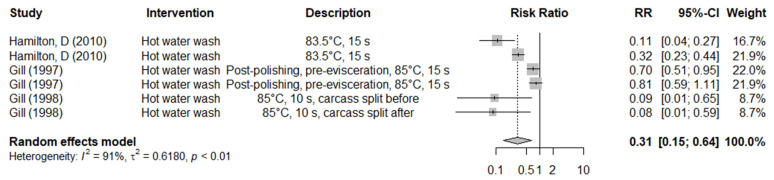
Forest plot of the results of combined controlled trials and before-and-after trials performed under commercial abattoir conditions to investigate the efficacy of hot water washing in reducing generic *E. coli* prevalence on pig carcasses [36,37,38].

**Figure 5:**
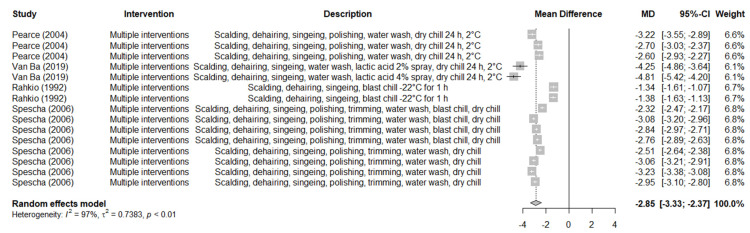
Forest plot of the results of before-and-after trials performed under commercial abattoir conditions to investigate the efficacy of multiple interventions in reducing aerobic colony count (log_10_ CFU) on pig carcasses [11,15,32,35].

For reasons of brevity, the results on the meta-analysis summary effects are shown below in tabulated form (Table 2, Table 3, Table 4 and Table 5). Furthermore, three examples of forest plots are also given (Figure 3, Figure 4 and Figure 5), and the remaining forest plots can be found in the Supplementary Materials. The results of the interventions for which there were not enough trials for a direct comparison of intervention effects are also presented in the Supplementary Materials.

#### 3.2.1. Preslaughter and Lairage Interventions

Regarding the investigated outcomes, no studies were identified that reported logistic slaughter, and only two studies reported lairage holding time [28] or misting pigs with disinfectant [29]. Six trials from one study found that *Enterobacteriaceae* counts in pig caecal content increased with an increase in both feed withdrawal time and lairage holding time (MD: 0.48, 95% CI: −0.10 to 1.06, *I*^2^ = 77%) [28]. Misting live pigs with disinfectant reduced *Enterobacteriaceae* counts on pig skin significantly when compared to water misting alone in only one trial (MD: −1.36, 95% CI: −2.91 to −0.19) [29].

#### 3.2.2. Standard Processing Procedures and GHP-Based Measures

Table 2 summarises the overall meta-analysis estimates of interventions’ effects for standard processing procedures and GHP-based measures such as scalding, dehairing, singeing, polishing, water washing, rectum sealing, alternative pluck removal and standard fat trimming.

Several studies investigated the efficacy of scalding in reducing indicator bacteria counts, with sufficient data to calculate meta-regression summary effects. Eight before-and-after trials showed that scalding under commercial abattoir conditions effectively reduced *Enterobacteriaceae* prevalence on pig carcasses (RR: 0.05, 95% CI: 0.02 to 0.12, *I*^2^ = 87%). In addition, 14 before-and-after trials from three studies showed that scalding significantly reduced ACC on pig carcasses by 2.84 log_10_ CFU/cm^2^ (MD: −2.84, 95% CI: −3.50 to −2.18, *I*^2^ = 99%). Another effective standard processing procedure for reducing *Enterobacteriaceae* prevalence and ACC on pig carcasses was singeing (RRL 0.25, 95% CI: 0.14 to 0.44, *I*^2^ = 90% and MD: −1.95, 95% CI: −2.40 to −1.50, *I*^2^ = 96%, respectively). In contrast, eight before-and-after trials investigating carcass water washing had a negligible effect in reducing *Enterobacteriaceae* prevalence (RR: 0.87, 95% CI: 0.80 to 0.94, *I*^2^ = 19%), while it increased the risk of carcass contamination with generic *E. coli* (RR: 1.09, 95% CI: 0.94 to 1.27, *I*^2^ = 26%). Water washing did not reduce ACC on pig carcasses as shown in 20 trials (MD: −0.12, 95% CI: −0.35 to 0.11, *I*^2^ = 90%).

Furthermore, rectum sealing, investigated in two studies with 18 controlled trials, effectively reduced the prevalence of *Y. enterocolitica* on pig carcasses (RR: 0.60, 95% CI: 0.41 to 0.89, *I*^2^ = 0%) (Figure 3). An alternative method with anal plugging prior to scalding and dehairing was investigated in only one study and reduced EBC around the anuses of plugged carcasses by 1.10 log CFU/cm^2^ compared with unplugged carcasses [30].

Expectedly, other standard processing procedures for carcasses, such as dehairing, polishing and standard fat trimming, were ineffective in reducing the prevalence or log counts of indicator bacteria and more often led to increase in contamination (Table 2). Dehairing increased ACC by 1.94 log_10_ CFU/cm^2^ (MD: 1.94, 95% CI: 1.67 to 2.11, *I*^2^ = 97%), while also significantly increasing the prevalence of *Enterobacteriaceae* (RR: 17.36, 95% CI: 6.88 to 43.75, *I*^2^ = 89%). Polishing at best did not change ACC or prevalence of *Enterobacteriaceae*, and similar results were reported with standard fat trimming (Table 2). One alternative pluck removal procedure, where the pluck set was partially removed, leaving the highly contaminated oral cavity, tonsils and tongue in place, did not meaningfully reduce the prevalence of *Y. enterocolitica*, *Enterobacteriaceae* and generic *E. coli*, and did not reduce ACC.

**Table 2:** A summary of the overall meta-analysis estimates of the interventions’ effects for standard processing procedures and good hygiene practices on pig carcasses.

Intervention	Microorganism ^a^	Study Design/ Conditions (No. of Studies/Trials) ^‡^	RR (95% CI) or MD (95% CI)	Heterogeneity *I^2^* (%) *	*p*-Value *	Reference(s)
Scalding	EBC	BA/Comm (1/8)	RR 0.05 (0.02, 0.12)	High (87%)	<0.01	[15]
Scalding	ACC	BA/Comm (4/14)	MD −2.48 (−3.50, −2.18)	High (99%)	0	[11,15,31,32]
Dehairing	EBC	BA/Comm (1/8)	RR 17.36 (6.88, 43.75)	High (89%)	<0.01	[15]
Dehairing	ACC	BA/Comm (3/12)	MD 1.94 (1.67, 2.21)	High (97%)	<0.01	[11,15,31]
Singeing	EBC	BA/Comm (1/4)	RR 0.25 (0.14, 0.44)	High (90%)	<0.01	[15]
Singeing	ACC	BA/Comm (3/9)	MD −1.95 (−2.4, −1.5)	High (96%)	<0.01	[11,15,32]
Polishing	EBC	BA/Comm (1/8)	RR 1.01 (0.8, 1.28)	High (86%)	<0.01	[15]
Polishing	ACC	BA/Comm (3/12)	MD 0.19 (−0.51, 0.89)	High (100%)	0	[11,14,15]
Water washing	ACC	CT_BA/Comm (4/20)	MD −0.12 (−0.35, 0.11)	High (90%)	<0.01	[14,15,31,33]
Water washing	EBC	BA/Comm (1/8)	RR 0.87 (0.8, 0.94)	Low (19%)	0.28	[15]
Water washing	Generic *E. coli*	BA/Comm (1/8)	RR 1.09 (0.94, 1.27)	Low (26%)	0.22	[33]
Rectum sealing	*Yersinia pseudotuberculosis*	CT/Comm (1/5)	RR 1.33 (0.24, 7.49)	Low (38%)	0.17	[12]
Rectum sealing	*Yersinia enterocolitica*	CT/Comm (2/18)	RR 0.6 (0.41, 0.89)	Low (0%)	0.88	[12,34]
Pluck removal	*EBC*	CT/Comm (1/3)	RR 0.98 (0.94, 1.03)	Low (0%)	0.56	[13]
Pluck removal	*Yersinia enterocolitica*	CT/Comm (1/3)	RR 0.33 (0.03, 3.18)	Low (0%)	1.00	[13]
Pluck removal	*Generic E. coli*	CT/Comm (1/3)	RR 0.87 (0.68, 1.11)	High (71%)	0.03	[13]
Pluck removal	*ACC*	CT/Comm (1/3)	MD −0.04 (−0.3, 0.21)	Low (34%)	0.22	[13]
Standard fat trimming	EBC	BA/Comm (1/8)	RR 1.16 (1.01, 1.33)	High (71%)	<0.01	[15]
Standard fat trimming	ACC	BA/Comm (1/8)	MD 0.06 (−0.16, 0.27)	High (95%)	<0.01	[15]

^‡^ CT—controlled trial; BA—before-and-after trial; Comm—commercial abattoir conditions. ^a^ ACC—aerobic colony count; EBC—*Enterobacteriaceae* count. * Homogenous trials: *p* > 0.05 on the test for heterogeneity; moderately heterogeneous: *p* < 0.05, *I*^2^ ≤ 60%; highly heterogeneous: *p* < 0.05, *I*^2^ > 60%.

#### 3.2.3. Prechilling Carcass Interventions

Data for only four hazard-based interventions for pig carcasses applied at the prechilling stage were available from the literature; interventions were hot water washing, lactic acid or acidified sodium chlorite (ASC) washing and novel pulsed light treatment (Table 3). Hot water washing investigated under commercial abattoir conditions significantly reduced the prevalence of generic *E. coli* on pig carcasses (RR: 0.31, 95% CI: 0.15 to 0.64, *I*^2^ = 91%) (Figure 4). It also significantly reduced ACC (MD: −1.32, 95% CI: −1.93 to −0.71, *I*^2^ = 93%) and generic *E. coli* count on pig carcasses (MD: −1.23, 95% CI: −1.89 to −0.57, *I*^2^ = 61%) (Table 3). Challenge trials conducted under laboratory conditions found that lactic acid wash reduced EBC by 0.72 log_10_ CFU/cm^2^ (MD: −0.72, 95% CI: −1.40 to −0.05, *I*^2^ = 98%) and ACC by 1.07 log_10_ CFU/cm^2^ (MD: −1.07, 95% CI: −1.33 to −0.81, *I*^2^ = 93%) on pig carcass meat. Another single study investigating prechilling lactic acid carcass spray efficacy after 24 h chilling found reductions of 0.49–1.05 log_10_ CFU/cm^2^ for ACC and of 0.73–1.38 log_10_ CFU/cm^2^ in generic *E. coli* count [35] (Supplementary Materials).

In 36 trials investigating pulsed light treatment, a significant reduction of 1.68 log_10_ CFU/cm^2^ in *Y. enterocolitica* on pig carcass meat (MD: −1.68, 95% CI: −1.99 to −1.37, *I*^2^ = 97%) was demonstrated. ASC wash was investigated in only one study with two trials; therefore, meta-analysis summary estimates were not calculated. However, two trials found RRs of 0.13 and 0.43 in reducing the prevalence of generic *E. coli* and mean reductions of 0.47–1.30 log_10_ CFU/cm^2^ for ACC and 1.05–1.64 log_10_ CFU/cm^2^ for generic *E. coli* count [36] (Supplementary Materials).

**Table 3:** A summary of the overall meta-analysis estimates of the interventions’ effects for pig carcass interventions: hot water washing, lactic acid washing and pulsed light treatment.

Intervention	Microorganism ^a^	Study Design/ Conditions (No. of Studies/Trials) ^‡^	RR (95% CI) or MD (95% CI)	Heterogeneity *I^2^* (%) *	*p*-Value *	Reference(s)
Hot water washing	Generic *E. coli*	CT_BA/Comm (3/6)	RR 0.31 (0.15, 0.64)	High (91%)	<0.01	[36,37,38]
Hot water washing	Generic *E. coli*	CT_BA/Comm (2/4)	MD −1.23 (−1.89, −0.57)	Moderate (61%)	0.05	[36,38]
Hot water washing	ACC	CT_BA/Comm (3/8)	MD −1.32 (−1.93, −0.71)	High (93%)	<0.01	[36,37,38]
Lactic acid washing	EBC	ChT/Lab (2/6)	MD −0.72 (−1.40, −0.05)	High (98%)	<0.01	[39,40]
Lactic acid washing	ACC	ChT/Lab (2/12)	MD −1.07 (−1.33, −0.81)	High (93%)	<0.01	[39,40]
Pulsed light treatment	*Yersinia enterocolitica*	ChT/Lab (1/36)	MD −1.68 (−1.99, −1.37)	High (97%)	<0.01	[41]

^‡^ CT—controlled trial; BA—before-and-after trial; ChT—challenge trial; Comm—commercial abattoir conditions. ^a^ ACC—aerobic colony count; EBC—*Enterobacteriaceae* count. * Homogenous trials: *p* > 0.05 on the test for heterogeneity; moderately heterogeneous: *p* < 0.05, *I*^2^ ≤ 60%; highly heterogeneous: *p* < 0.05, *I*^2^ > 60%.

#### 3.2.4. Chilling

Three different methods of chilling were studied: conventional dry, blast and water spray chilling. Conventional dry chilling produced more consistent reductions in indicator bacteria counts, whereas other methods of chilling, such as combination of blast and conventional chilling, produced mixed results (Table 4). In four before-and-after trials under commercial conditions, conventional dry chilling effectively reduced *Enterobacteriaceae* prevalence on pig carcasses (RR: 0.32, 95% CI: 0.21 to 0.48, *I*^2^ = 81%). Likewise, fifteen before-and-after trials showed a small but significant 0.36 log_10_ CFU/cm^2^ reduction in ACC (MD: −0.36, 95% CI: −0.61 to −0.12, *I*^2^ = 94%). Conventional chilling also significantly reduced ACC (MD: −1.77, 95% CI: −2.54 to −1.01, *I*^2^ = 35%) and generic *E. coli* count (MD: −2.44, 95% CI: −3.93 to −0.95, *I*^2^ = 89%) in four challenge laboratory trials on pig carcass meat.

Blast chilling followed by conventional dry chilling reduced prevalence of *Enterobacteriaceae* on pig carcasses (RR: 0.10, 95% CI: 0.02 to 0.47, *I*^2^ = 78%), but not the prevalence of generic *E. coli* (RR: 0.61, 95% CI: 0.34 to 1.11, *I*^2^ = 50%) or ACC (MD: −0.17, 95% CI: −0.47 to 0.12, *I*^2^ = 93%) in four before-and-after trials conducted under commercial abattoir conditions. In four challenge trials, blast chilling produced similar reduction effects as conventional dry chilling for ACC (MD: −1.70, 95% CI: −2.81 to −0.59, *I*^2^ = 57%) and generic *E. coli* count (MD: −2.64, 95% CI: −4.56 to −0.73, *I*^2^ = 94%) on pig carcass meat.

Blast chilling followed by water spray chilling largely did not reduce the prevalence of *Enterobacteriaceae* (RR: 0.55, 95% CI: 0.34 to 0.90, *I*^2^ = 46%) and actually led to increased ACC (MD: 0.01, 95% CI: −1.00 to 2.22, *I*^2^ = 88%) on pig carcass meat in trials conducted under commercial abattoir conditions.

**Table 4:** A summary of the overall meta-analysis estimates of the interventions’ effects for different chilling methods on pig carcasses.

Intervention	Microorganism ^a^	Study Design/ Conditions (No. of Studies/Trials) ^‡^	RR (95% CI) or MD (95% CI)	Heterogeneity *I^2^* (%) *	*p*-Value *	Reference(s)
Conventional dry chilling	EBC	BA/Comm (1/4)	RR 0.32 (0.21, 0.48)	High (81%)	<0.01	[15]
Conventional dry chilling	ACC	BA/Comm (4/15)	MD −0.36 (−0.61, −0.12)	High (94%)	<0.01	[11,15,33,42]
Blast and conventional chilling	EBC	BA/Comm (1/4)	RR 0.1 (0.02, 0.47)	High (78%)	<0.01	[15]
Blast and conventional chilling	Generic *E. coli*	BA/Comm (1/4)	RR 0.61 (0.34, 1.11)	Low (50%)	0.11	[33]
Blast and conventional chilling	ACC	BA/Comm (3/10)	MD −0.17 (−0.47, 0.12)	High (93%)	<0.01	[15,32,33]
Blast and water spray chilling	EBC	BA/Comm (2/3)	RR 0.55 (0.34, 0.9)	Low (46%)	0.16	[33,43]
Blast and water spray chilling	ACC	BA/Comm (2/3)	MD 0.01 (−1.0, 2.22)	High (88%)	<0.01	[33,43]
Blast chilling	Generic *E. coli*	ChT/Lab (1/4)	MD −2.64 (−4.56, −0.73)	High (94%)	<0.01	[44]
Blast chilling	ACC	ChT/Lab (1.4)	MD −1.7 (−2.81, −0.59)	Low (57%)	0.07	[44]
Blast vs conventional chilling	ACC	ChT/Lab (1.4)	MD −0.04 (−1.02, 0.94)	Low (30%)	0.23	[44]
Conventional dry chilling	ACC	ChT/Lab (1/4)	MD −1.77 (−2.54, −1.01)	Low (35%)	0.20	[44]
Conventional dry chilling	Generic *E. coli*	ChT/Lab (1/4)	MD −2.44 (−3.93, −0.95)	High (89%)	<0.01	[44]

^‡^ BA—before-and-after trial; ChT—challenge trial; Comm—commercial abattoir conditions. ^a^ ACC—aerobic colony count; EBC—*Enterobacteriaceae* count. * Homogenous trials: *p* > 0.05 on the test for heterogeneity; moderately heterogeneous: *p* < 0.05, *I*^2^ ≤ 60%; highly heterogeneous: *p* < 0.05, *I*^2^ > 60%.

#### 3.2.5. Multiple Interventions

Several studies conducted under commercial abattoir conditions investigated the effects of multiple interventions sequentially applied on the slaughterline. The majority of these trials investigated the efficacy of sequential use of scalding, dehairing, singeing, polishing, trimming, water washing (with or without prechilling lactic acid spray) and blast and/or dry chilling (Table 5). Eight before-and-after trials investigating multiple interventions showed they effectively reduced *Enterobacteriaceae* prevalence on pig carcasses (RR: 0.11, 95% CI: 0.05 to 0.23, *I*^2^ = 94%). Similarly, another fifteen before-and-after trials found significant reductions (2.85 log_10_ CFU/cm^2^) of ACC on pig carcasses (MD: −2.85, 95% CI: −3.33 to −2.37, *I*^2^ = 97%) (Figure 5). In only one study/trial that investigated the sequential use of scalding, dehairing, singeing and scraping, reductions achieved for ACC, EBC and generic *E. coli* were 0.87 log_10_ CFU/cm^2^, 2.15 log_10_ CFU/cm^2^ and 2.20 log_10_ CFU/cm^2^, respectively [31].

**Table 5:** A summary of the overall meta-analysis estimates of the multiple intervention effects on pig carcasses.

Intervention	Microorganism ^a^	Study Design/ Conditions (No. of Studies/Trials) ^‡^	RR (95% CI) or MD (95% CI)	Heterogeneity *I^2^* (%) *	*p*-Value *	Reference(s)
Multiple **	EBC	BA/Comm (1/8)	RR 0.11 (0.05, 0.23)	High (94%)	<0.01	[15]
Multiple ***	ACC	BA/Comm (4/15)	MD −2.85 (−3.33, −2.37)	High (97%)	<0.01	[11,15,32,35]

^‡^ BA—before-and-after trial; Comm—commercial abattoir conditions. ^a^ ACC—aerobic colony count; EBC—*Enterobacteriaceae* count. * Homogenous trials: *p* > 0.05 on the test for heterogeneity; moderately heterogeneous: *p* < 0.05, *I*^2^ ≤ 60%; highly heterogeneous: *p* < 0.05, *I*^2^ > 60%. ** Interventions including scalding, dehairing, singeing, polishing, trimming, water washing and blast and/or dry chilling. *** Interventions including scalding, dehairing, singeing, polishing, water washing and/or lactic acid washing and blast/dry chilling.

## 4. Discussion

The aim of this study was to analyse a range of abattoir interventions and to identify those that have a significant reduction effect on microorganisms of concern (i.e., indicator bacteria and *Yersinia* spp.) using the statistical power of a meta-analysis tool. Overall, 30 years of literature were reviewed, and following a structured and stringent review process, 22 articles were found eligible to conduct a meta-analysis. The final outcomes were 48 forest plots and 40 meta-analysis summary effects generated. Data were included for interventions from the preslaughter stage (i.e., lairage holding time, feed withdrawal and misting pigs with disinfectant), standard processing procedures for pig carcasses, hazard-based prechilling interventions and multiple carcass interventions, to the final chilling stage. Despite the fact that this systematic review included such a large body of literature and investigated interventions for four microorganisms, the main findings and concerns are that pig interventions are not a well-researched area, and there are significant gaps in the literature. Furthermore, even when some studies existed for a given intervention/outcome, more than half of identified eligible studies either did not report measures of variability, which are essential for meta-analysis or had data presented in difficult-to-extract graph format. In line with the problems with methodological study design in some of the articles reviewed, the data reporting was a significant obstacle in obtaining more useful data for analysis purpose. Among 40 pooled meta-analysis summary effects (pooled risk ratios (RRs), for prevalence outcomes, or pooled log mean difference for concentration outcomes), only 13 were with low or moderate heterogeneity (and, therefore, we had better confidence in the results). Meta-analysis is a useful analytical tool for combining the results of multiple primary research studies into a weighted, average estimate for, in our case, intervention effect. The limitation of this analysis could be that, even though every effort was made to stratify data into the most similar subgroups, sometimes within-subgroup data likely resulted from studies/trials with recorded or unrecorded differences. This stratification approach was chosen for pragmatic reasons to combine a sufficient number of trials for meta-analysis, wherever it was possible, from a limited pool of data. As a consequence, details about intervention application parameters (e.g., acid concentration, temperature, duration) and differences between study sampling and laboratory methods were not investigated as possible sources of variation in intervention effects across studies. These and other study factors could well contribute to the heterogeneity in effects observed for many intervention categories, but it was beyond the scope of this systematic review and meta-analysis to investigate these factors in detail. However, the created forest plots contain sufficient information and description about analysed interventions. Overall, this systematic review clearly has identified a lack of large, controlled trials conducted under commercial conditions, with sound study design and adequate reporting of intervention protocols. This was particularly case with hazard-based, prechilling interventions at slaughter, and particularly for *Yersinia* spp. This was surprising given that carriage of *Yersinia enterocolitica* on pig tonsils and prevalence on pig carcasses at slaughter can be as high as 90% and 60%, respectively, and up to 30% in raw pork [7]. Inadequate reporting of protocols, lack of addressing any confounders, inappropriate choice of outcome measurement units when expected microbial counts were low and faults in reporting of results (e.g., lack of measures of variability) were common and reduced further the already sparse pool of scientific data in this area.

The microbial status of pig carcasses on the slaughterline depends on many factors, including preslaughter hygiene and animal cleanliness. Stress factors during transport and lairage can provoke the shedding of bacteria, including pathogens, increasing the risk of faecal contamination of carcasses during slaughter [3,28]. Lairage time and direct or indirect contact of groups of pigs during lairaging prior to slaughter influence the bacterial load of carcasses or the occurrence of pathogens in lymphoid tissues [45,46]. For example, a higher prevalence of *Y. enterocolitica* was found in the tonsils of pigs slaughtered in the slaughterhouses where pigs were held in the lairage pens separated by a fence that allowed contact between the pigs (40% and 52%), than in the tonsils of pigs slaughtered in the slaughterhouse that had a solid wall between lairage pens thus preventing contact between pigs (29%) [47]. Moreover, in one study, a higher prevalence of *Salmonella* was found in pigs in the lairage than in the farm of origin [6]. Considering the outcomes included in the present systematic review, only one cohort study on lairage interventions with six trials was eligible to conduct a meta-analysis [28]. This showed that feed withdrawal time and holding time in lairage have no significant effect on *Enterobacteriaceae* count in pig caecal content and likely no effect in their further spread on the slaughterline during slaughter and dressing. However, the lairage is known to be a source of contamination with *Salmonella* [48]; thus, it is important that further research is conducted to assess effective ways to reduce contamination before pigs enter the slaughterline.

Standard processing procedures and good hygienic practices in pig slaughtering are designed to maintain high levels of hygiene and produce final carcasses with low microbial load. Various slaughter operations affect the bacterial status of pig skin, offal and carcasses in a positive or negative way, i.e., they can increase contamination or reduce the microbial load [1]. Thermal treatments are a well-known hurdle used to reduce bacterial contamination and are used to varying degrees in pig carcass scalding (with warm water), singeing (open-flame gas burning) or spraying/washing (hot water, steam). The present meta-analysis identified that within standard processing procedures, scalding and singeing were the most effective in reducing *Enterobacteriaceae* prevalence and ACC on pig carcasses (by around 2 logs). Although their primary purpose is dehairing, they contribute to the reduction of microbial contamination of pig carcasses [1]. Scalding time and temperatures vary from abattoir to abattoir, and differences in these parameters produce different reductions of microbial contamination [15,31]. The current meta-analysis found dehairing and polishing, on the other hand, increased the counts and/or prevalence of aerobic bacteria and *Enterobacteriaceae*, as expected. Dehairing machines are always contaminated with bacteria and are washed with recirculated hot water only. A recent study reported that recycled water in the dehairing process is the main source of contamination of pig carcasses with *Salmonella* at the abattoir [49]. It is also generally accepted that subsequent polishing facilitates the redistribution of any surviving bacteria from the singeing process throughout the pig carcass [11]. In this meta-analysis, we found that polishing at best did not change ACC or prevalence of *Enterobacteriaceae*. Other GHP measures investigated provided mixed results. For example, water washing only negligibly reduced the prevalence of *Enterobacteriaceae* and ACC, and slightly increased prevalence of generic *E. coli*. Furthermore, combined effects of sequential use of several standard processing procedures (scalding, dehairing, singeing and scraping) achieved reductions in ACC, EBC and generic *E. coli* counts of up to 2 logs, although only one study/trial was eligible for this meta-analysis [31]. This was expected, as usually two or more interventions applied sequentially produce a larger effect than any individual intervention [16].

The evisceration procedure on the slaughterline is one of the most critical steps, which begins with the loosening and sealing of the rectum. The general purpose of this hygienic procedure is to avoid faecal contamination of the carcass and organs. Data analysis showed its efficacy in reducing *Y. enterocolitica* prevalence on carcasses, suggesting that this procedure should always be applied. In addition, the data analysis revealed strong evidence, derived from laboratory trials, of the efficacy of pulsed light to reduce *Y. enterocolitica* on pig carcasses. Pathogenic *Y. enterocolitica* is a priority hazard to control in pork production and more data are needed for its effective control in the meat chain [50,51]. The present systematic review did not identify any other published studies investigating other potentially relevant interventions to reduce *Y. enterocolitica* on carcasses. Thus, the effectiveness of interventions in reducing *Y. enterocolitica* on pig carcasses is an insufficiently researched area, and there is a serious lack of data in this respect.

In some abattoirs, carcass interventions are used with the aim of reducing bacterial loads and the carriage of pathogens detected at farm level. This includes hazard-based interventions, such as hot water washing. Combinations of controlled and before-and-after trials conducted under commercial abattoir conditions showed hot water washing effectively reduces the prevalence and counts of generic *E. coli* and ACC, by around 1 log. Hot water washing is also a very effective intervention commonly used for beef carcasses [16]. In Denmark, hot water washing is used on pig carcasses from batches originating from *Salmonella*-positive pig herds. It has been found to be more cost-effective than steam vacuum and lactic acid washing [52,53].

Lactic acid washing also significantly reduced EBC and ACC on pig carcass meat, but data eligible for meta-analysis came only from studies investigated under laboratory conditions. EFSA in 2018 issued a scientific opinion on the safety and efficacy of organic acids for pig carcasses [54]. In its review, EFSA found that spraying pig carcasses with lactic acid (2–5% solutions at temperatures of up to 80 °C) prior to chilling is of no safety concern (provided that the substances comply with the EU specifications for food additives) and was efficacious compared to untreated control. However, EFSA could not conclude whether lactic acid was more efficacious than water treatment when pig carcasses were sprayed at the prechilling stage. EFSA’s review was systematic in nature and included 11 literature sources (16 eligible experiments) on lactic acid but without meta-analysis. Some of analysed literature sources were on pork meat cuts post-chill or ground pork (therefore, these were excluded from our study), and some of them did not report measures of variability that are needed for meta-analysis. Following a similar positive EFSA opinion from 2011 [55], lactic acid was permitted for use in EU abattoirs (Regulation EC 101/2013) for beef carcass washing [56]. Lactic acid washes are efficacious interventions for beef carcasses, usually reducing indicator bacteria counts by 1–1.5 logs under commercial abattoir conditions [16]. Studies investigating other organic acids (e.g., acetic acid) and other chemical agents for pig carcass washes were lacking or did not meet criteria for this meta-analysis.

Chilling is a procedure mandated by the legislation, and there are several methods of chilling with varying degrees of effectiveness with regard to reducing microbial contamination. Usually, dressed pig carcasses are blasted with air at approximately −8 °C to −20 °C for up to 1 h to quickly reduce carcass temperature, and then the carcasses are transferred to a conventional chiller at approximately 2 °C for the remaining chilling time. Studies focusing on the effects of a combination of blast chilling followed by conventional chilling and/or each individual chilling method showed inconsistent results [15,33]. It is likely that the effectiveness of these interventions is influenced by temperature, air velocity, humidity, and duration [33]. Furthermore, it is likely that some microbial reductions are due to inactivation due to surface drying but also due to reduced viability of bacteria to recover from chilling for subsequent growth and/or inability of swabbing method to pick up bacteria cells from the dry surface. These factors hinder microbial detection, and therefore, proper study design and using specific media to enable microbial recovery is necessary when investigating the efficacy of chilling.

Multiple interventions when applied sequentially (scalding, dehairing, singeing, polishing, trimming, water washing (with or without prechilling lactic acid spray) and blast and/or dry chilling) produced the biggest reductions of up to 3 logs of ACC and significantly reduced the prevalence of *Enterobacteriaceae* on pig carcasses. Application of multiple slaughterline interventions is expected to improve the overall microbial status of carcasses and reduce risks further than do single interventions [16], particularly when they are extended in an overall multiple-hurdle strategy with decontamination of resulting portioned meat and pork trimmings [57]. Furthermore, use of interventions is necessary in high risk situations (e.g., when an abattoir is unable to sufficiently reduce risks arising from specific farms/animal batches by using process hygiene alone), to meet the targets on chilled carcasses [16,53]. As such, pig interventions at abattoir stage (preslaughter and slaughter) form an essential component of the meat safety assurance system.

## 5. Conclusions

This systematic review and meta-analysis were performed to assess the effectiveness of abattoir interventions in reducing indicator bacteria counts (i.e., ACC, EBC and generic *E. coli* count) and the count and/or prevalence of *Yersinia* spp. on pig carcasses. There were noticeable gaps in the literature spanning 30 years on studies investigating pig interventions. This was very clear, particularly with respect to interventions with proven efficacy in some other meat species (e.g., beef carcasses), such as carcass steam pasteurisation and organic acid washes (acetic acid and lack of data on lactic acid), and there is a distinct lack of sufficient data on hot water washing and blast chilling. Several commercial trials found that common standard processing procedures, such as scalding and singeing, are very effective in reducing indicator bacteria counts. This meta-analysis found that pig carcass scalding effectively reduces the prevalence of *Enterobacteriaceae* (RR 0.05) and ACC (2.84 log_10_ CFU/cm^2^), as does singeing (RR: 0.25, and 1.95 log_10_ CFU/cm^2^, respectively). Rectum sealing effectively reduces the prevalence of *Y. enterocolitica* on pig carcasses (RR 0.60). A multiple hurdle approach that included the sequential application of carcass interventions significantly reduces *Enterobacteriaceae* prevalence (RR: 0.11) and ACC on carcasses (2.85 log_10_ CFU/cm^2^). Nevertheless, most of the data were generated from highly heterogeneous studies and trials, likely due to the inherent differences between studies, but also from the small number of studies/trials eligible for this meta-analysis. This indicates that better designed research, with results presented numerically and with measures of variability, is needed. This is particularly the case for *Y. enterocolitica*, which is a priority pathogen for control in the pig meat chain. Overall, the results suggest that scalding, singeing, washing with hot water and/or lactic acid, and dry chilling effectively reduce the counts of indicator bacteria on pig carcasses. The meta-analysis also found evidence that pathogenic *Y. enterocolitica* on pig carcasses is effectively reduced by the standard procedure of rectum sealing; however, this was the only intervention for *Yersinia* investigated under commercial conditions. All these effective interventions should be recommended for commercial use in abattoirs and should form an essential part of integrated pig meat controls. Furthermore, the data generated in this meta-analysis can be used for further modelling and risk assessment work and for providing recommendations on the use of specific interventions in pig abattoirs.

## Figures and Tables

**Table 1:** Key characteristics of 25 relevant articles on pig interventions.

Article Characteristic	Number of Articles ^1^	%
**Region**		
North America	6	24%
Europe	16	64%
Australia/South Pacific	1	4%
Asia/Middle East	2	8%
Central and South America/Caribbean	0	0
Africa	0	0
**Document type**		
Journal article	25	100%
Thesis	0	0
Conference paper	0	0
Government or research report	0	0
**Study design**		
Challenge trial	7	24.1%
Before-and-after trial	12	41.4%
Controlled trial	9	31%
Cohort study	1	3.4%
**Study conditions**		
Laboratory conditions	6	23.1%
Commercial abattoir conditions	18	69.2%
Research/pilot plant	2	7.7%
**Intervention category/subcategory**		
Pig handling in lairage	2	5.1%
Scalding	4	10.3%
Singeing	4	10.3%
Other standard processing procedures/GHP	8	20.5%
Carcass prechilling interventions	12	30.8%
Chilling, spray chilling, blast chilling	9	23.1%
**Outcomes investigated**		
Aerobic colony count	17	37.7%
*Enterobacteriaceae* count/prevalence	9	20.0%
Generic *E. coli* count/prevalence	12	26.6%
*Yersinia enterocolitica* count/prevalence	6	13.3%
*Yersinia pseudotuberculosis* prevalence	1	2.2%
**Risk-of-bias concerns**		
Low	22	88%
Some concerns	2	8%
High	1	4%

^1^ Although the number of included articles was 25, the number of articles per category may not be equal, as often studies incorporated more than one study condition and/or intervention category and investigated multiple outcomes.

## Data Availability

Data is contained within the article.

## Supplementary Materials

The following supporting information can be downloaded at: https://www.mdpi.com/article/10.3390/foods11142110/s1, Database search results; Detailed systematic review protocols; List of references for studies used in meta-analysis; Examples of intervention forest plots.
