# Supplementary material for: Systematic Review and Meta-Analysis of the Efficacy of Interventions Applied during Primary Processing to Reduce Microbial Contamination on Pig Carcasses
Source: Foods. 2022 Jul 15;11(14):2110. doi: 10.3390/foods11142110 (PMC9315615; doi:10.3390/foods11142110)
Supplement: Supplementary file 1 [file foods-11-02110-s001.zip › foods-1688196-supplementary.pdf]

foods MDPI

# Supplementary Material

## Database searches

| | |
|---|---|
| Date | 03/12/2020 |
| Performed by | Dragan Antic |
| Database | Scopus |
| Search string: | *(salmonella OR salmonellae OR yersinia OR aerob* OR enterobacteriaceae OR "escherichia coli" OR "e. coli") AND (intervention* OR decontaminat** OR contamination *OR treatment* OR inactiv* OR reduce* OR reducing OR reduction OR efficacy OR cleaning OR disinfect* OR lairage* OR slaughter* OR hygien* OR HACCP OR **carcas* OR** scald* OR dehair* OR skin* OR singe* OR eviscerat* OR bung* OR wash* OR rins* OR spray* OR vacuum* OR steam OR trim* OR pasteuriz* OR pasteuris* OR "hot water" OR chlorine OR "organic acid*" OR "lactic acid" OR chill* OR "blast chilling" OR "ultra-rapid chilling" OR "ultra rapid chilling") AND (swine OR pig OR pigs OR sow OR sows OR hog OR boar OR porcine OR pork)*<br><br>in Article title<br>OR<br>in Abstract<br>OR<br>in Key words |
| Limits | 1990-2021 |
| Hits | 9,146 |

| | |
|---|---|
| Date | 03/12/2020 |
| Performed by | Dragan Antic |
| Database | CAB Direct |
| Search string: | *(salmonella OR salmonellae OR yersinia OR aerob* OR enterobacteriaceae OR "escherichia coli" OR "e. coli") AND (intervention* OR decontaminat* OR contamination OR treatment* OR inactiv* OR reduce* OR reducing OR reduction OR efficacy OR cleaning OR disinfect* OR lairage* OR slaughter* OR hygien* OR HACCP OR carcas* OR scald* OR dehair* OR skin* OR singe* OR eviscerat* OR bung* OR wash* OR rins* OR spray* OR vacuum* OR steam OR trim* OR pasteuriz* OR pasteuris* OR "hot water" OR chlorine OR "organic acid*" OR "lactic acid" OR chill* OR "blast chilling" OR "ultra-rapid chilling" OR "ultra rapid chilling") AND (swine OR pig OR pigs OR sow OR sows OR hog OR boar OR porcine OR pork)*<br><br>in All fields |
| Limits | 1990-2021 |
| Hits | 8,019 |

| | |
|---|---|
| Date | 03/12/2020 |
| Performed by | Kurt Houf |
| Database | SciELO |
| Search string: | *(salmonella OR salmonellae OR yersinia OR aerob* OR enterobacteriaceae OR "escherichia coli" OR "e. coli") AND (intervention* OR decontaminat* OR contamination OR treatment* OR inactiv* OR reduce* OR reducing OR reduction OR efficacy OR cleaning OR disinfect* OR lairage* OR slaughter* OR hygien* OR HACCP OR carcas* OR scald* OR dehair* OR skin* OR singe* OR eviscerat* OR bung* OR wash* OR rins* OR spray* OR vacuum* OR steam OR trim* OR pasteuriz* OR pasteuris* OR "hot water" OR chlorine OR "organic acid*" OR "lactic acid" OR chill* OR "blast chilling" OR "ultra-rapid chilling" OR "ultra rapid chilling") AND (swine OR pig OR pigs OR sow OR sows OR hog OR boar OR porcine OR pork)*<br><br>in Article title<br>OR<br>in Abstract<br>OR<br>in Key words |
| Limits | 2002-2021 |
| Hits | 169 |

**<u>List of relevant articles used to improve pre-test search algorithms in the databases, such that these articles would be identified</u>**

**Lairage interventions**

Larsen, S. T., McKean, J. D., Hurd, H. S., Rostagno, M. H., Griffith, R. W., & Wesley, I. V. (2003). Impact of commercial preharvest transportation and holding on the prevalence of *Salmonella enterica* in cull sows. *Journal of food protection*, 66(7), 1134-1138.

Argüello, H., Carvajal, A., Álvarez-Ordóñez, A., Jaramillo-Torres, H. A., & Rubio, P. (2014). Effect of logistic slaughter on *Salmonella* contamination on pig carcasses. *Food research international*, 55, 77-82.

Mannion C., Fanning, J., McLernon, J., Lendrum, L., Gutierrez, M., Duggan, S., & Egan, J. (2011). The role of transport, lairage and slaughter processes in the dissemination of *Salmonella* spp. in pigs in Ireland. *Food Research International*, 45, 871.

Walia, K., Lynch, H., Grant, J., Duffy, G., Leonard, F. C., Lawlor, P. G., & Gardiner, G. E. (2017). The efficacy of disinfectant misting in the lairage of a pig abattoir to reduce *Salmonella* and *Enterobacteriaceae* on pigs prior to slaughter. *Food Control*, 75, 55-61.

Swanenburg, M., Van der Wolf, P. J., Urlings, H. A. P., Snijders, J. M. A., & Van Knapen, F. (2001). *Salmonella* in slaughter pigs: the effect of logistic slaughter procedures of pigs on the prevalence of *Salmonella* in pork. *International Journal of Food Microbiology*, 70(3), 231-242.

**Carcass interventions**

Trivedi, S., Reynolds, A. E., & Chen, J. (2007). Use of a commercial household steam cleaning system to decontaminate beef and hog carcasses processed by four small or very small meat processing plants in Georgia. *Journal of food protection*, 70(3), 635-640.

Carpenter, C. E., Smith, J. V., & Broadbent, J. R. (2011). Efficacy of washing meat surfaces with 2% levulinic, acetic, or lactic acid for pathogen decontamination and residual growth inhibition. *Meat science*, 88(2), 256-260.

Smulders, F. J., Wellm, G., Hiesberger, J., Bauer, A., & Paulsen, P. (2012). The potential of the combined application of hot water sprays and steam condensation at subatmospheric pressure for decontaminating inoculated pig skin and muscle surfaces. *Food control*, 24(1-2), 154-159.

Alban, L., & Sørensen, L. L. (2010). Hot-water decontamination is an effective way of reducing risk of *Salmonella* in pork. *Fleischwirtschaft*, 90(9), 109-113.

Van Ba, H., Seo, H. W., Seong, P. N., Kang, S. M., Cho, S. H., Kim, Y. S., ... & Kim, J. H. (2019). The fates of microbial populations on pig carcasses during slaughtering process, on retail cuts after slaughter, and intervention efficiency of lactic acid spraying. *International journal of food microbiology*, 294, 10-17.

**Standard processing procedures / Good hygiene practices**

Biasino, W., De Zutter, L., Woollard, J., Mattheus, W., Bertrand, S., Uyttendaele, M., & Van Damme, I. (2018). Reduced contamination of pig carcasses using an alternative pluck set removal procedure during slaughter. *Meat science*, 145, 23-30.

Sanchez-Rodríguez, J. A., Navas, L., Vinuesa, F. M., Castells, C., Martínez, M. A., Lopez, A., ... & Cabrera-Vique, C. (2018). New insights on the risk factors associated with the presence of *Salmonella* on pig carcasses. Lessons from small slaughterhouses. *Food Control*, 87, 46-52.

Gill, C. O., Dussault, F. H. R. A., Holley, R. A., Houde, A., Jones, T., Rheault, N., ... & Quessy, S. (2000). Evaluation of the hygienic performances of the processes for cleaning, dressing and cooling pig carcasses at eight packing plants. *International Journal of Food Microbiology*, 58(1-2), 65-72.

Spescha, C., Stephan, R., & Zweifel, C. (2006). Microbiological contamination of pig carcasses at different stages of slaughter in two European Union-approved abattoirs. *Journal of food protection*, 69(11), 2568-2575.

Pearce, R.A., Bolton, D.J., Sheridan, J.J., McDowell, D.A., Blair, I.S. & Harrington, D. (2004). Studies to determine the critical control points in pork slaughter hazard analysis and critical control point systems. *International Journal of Food Microbiology*, 90(3): 331–339.

**Chilling**

Nesbakken, T., Eckner, K., & Røtterud, O. J. (2008). The effect of blast chilling on occurrence of human pathogenic *Yersinia enterocolitica* compared to *Campylobacter* spp. and numbers of hygienic indicators on pig carcasses. *International journal of food microbiology*, 123(1-2), 130-133.

Chang, V. P., Mills, E. W., & Cutter, C. N. (2003). Reduction of bacteria on pork carcasses associated with chilling method. *Journal of food protection*, 66(6), 1019-1024.

Bolton, D. J., Pearce, R. A., Sheridan, J. J., Blair, I. S., McDowell, D. A., & Harrington, D. (2002). Washing and chilling as critical control points in pork slaughter hazard analysis and critical control point (HACCP) systems. *Journal of Applied Microbiology*, 92(5), 893-902.

Barron, U. G., Bergin, D., & Butler, F. (2008). A meta-analysis study of the effect of chilling on prevalence of *Salmonella* on pig carcasses. *Journal of food protection*, 71(7), 1330-1337.

Lenahan, M., Crowley, H., O'brien, S. B., Byrne, C., Sweeney, T., & Sheridan, J. J. (2009). The potential use of chilling to control the growth of *Enterobacteriaceae* on porcine carcasses and the incidence of *E. coli* O157: H7 in pigs. *Journal of applied microbiology*, 106(5), 1512-1520.

**<u>List of search verification articles with reference lists that were searched by our review team members</u>**

**<u>Review articles:</u>**

Arguello, H., Alvarez-Ordonez, A., Carvajal, A., Rubio, P. & Prieto, M. (2013). Role of slaughtering in *Salmonella* spreading and control in pork production. *Journal of Food Protection*, 76(5): 899–911.

Baer, A. A., Miller, M. J., & Dilger, A. C. (2013). Pathogens of interest to the pork industry: a review of research on interventions to assure food safety. *Comprehensive Reviews in Food Science and Food Safety*, 12(2), 183-217.

Buncic, S., & Sofos, J. (2012). Interventions to control *Salmonella* contamination during poultry, cattle and pig slaughter. *Food research international*, 45(2), 641-655.

Dahl, J. (2013). Controlling *Salmonella* in live pigs and at slaughter: the Danish experience. *Veterinary journal*, 197(3), 529.

De Busser, E. V., De Zutter, L., Dewulf, J., Houf, K., & Maes, D. (2013). *Salmonella* control in live pigs and at slaughter. *The Veterinary Journal*, 196(1), 20-27.

EFSA (2018). Evaluation of the safety and efficacy of the organic acids lactic and acetic acids to reduce microbiological surface contamination on pork carcasses and pork cuts. *EFSA Journal*, 16(12), 5482.

Laukkanen-Ninios, R., Fredriksson-Ahomaa, M., Korkeala, H., 2014. Enteropathogenic *Yersinia* in the pork production chain: challenges for control. *Comprehensive Reviews in Food Science and Food Safety* 13, 1165-1191.

Loretz, M., Stephan, R., & Zweifel, C. (2011). Antibacterial activity of decontamination treatments for pig carcasses. *Food Control*, 22(3-4), 1121-1125.

Nesbakken, T. (2015). Update on *Yersinia* as a foodborne pathogen: Analysis and control. In *Advances in Microbial Food Safety* (Vol. 2, pp. 33-58).

FAO (2016). Interventions for the Control of Non-typhoidal *Salmonella* Spp. in Beef and Pork: Meeting Report and Systematic Review: Food and Agriculture Organization of the United Nations.

Belluco, S., Barco, L., Roccato, A., Ricci, A., 2015. Variability of *Escherichia coli* and *Enterobacteriaceae* counts on pig carcasses: A systematic review. *Food Control* 55, 115-126.

Gonzales Barron, U., Bergin, D., Butler, F., 2008. A meta-analysis study of the effect of chilling on prevalence of *Salmonella* on pig carcasses. *Journal of Food Protection* 71, 1330-1337.

O'Connor, A. M., Wang, B., Denagamage, T., & McKean, J. (2012). Process mapping the prevalence of *Salmonella* contamination on pork carcass from slaughter to chilling: a systematic review approach. *Foodborne pathogens and disease*, 9(5), 386-395.

Totton, S.C., Glanville, J.M., Dzikamunhenga, R.S., Dickson, J.S., O'Connor, A.M., 2016. Systematic review of the magnitude of change in prevalence and quantity of *Salmonella* after administration of pathogen reduction treatments on pork carcasses. *Animal Health Research Reviews* 17, 39-59.

Wilhelm, B.J., Young, I., Cahill, S., Desmarchelier, P., Nakagawa, R., Rajić, A., 2017. Interventions to reduce non-typhoidal *Salmonella* in pigs during transport to slaughter and lairage: Systematic review, meta-analysis, and research synthesis based infection models in support of assessment of effectiveness. *Preventive Veterinary Medicine* 145, 133-144.

**<u>Primary research articles</u>:**

Mannion C., Fanning, J., McLernon, J., Lendrum, L., Gutierrez, M., Duggan, S., & Egan, J. (2011). The role of transport, lairage and slaughter processes in the dissemination of *Salmonella* spp. in pigs in Ireland. *Food Research International*, 45, 871.

Walia, K., Lynch, H., Grant, J., Duffy, G., Leonard, F. C., Lawlor, P. G., & Gardiner, G. E. (2017). The efficacy of disinfectant misting in the lairage of a pig abattoir to reduce *Salmonella* and *Enterobacteriaceae* on pigs prior to slaughter. *Food Control*, 75, 55-61.

Argüello, H., Carvajal, A., Álvarez-Ordóñez, A., Jaramillo-Torres, H. A., & Rubio, P. (2014). Effect of logistic slaughter on *Salmonella* contamination on pig carcasses. *Food research international*, 55, 77-82.

Alban, L., & Sørensen, L. L. (2010). Hot-water decontamination is an effective way of reducing risk of *Salmonella* in pork. *Fleischwirtschaft*, 90(9), 109-113.

Carpenter, C. E., Smith, J. V., & Broadbent, J. R. (2011). Efficacy of washing meat surfaces with 2% levulinic, acetic, or lactic acid for pathogen decontamination and residual growth inhibition. *Meat science*, 88(2), 256-260.

Biasino, W., De Zutter, L., Woollard, J., Mattheus, W., Bertrand, S., Uyttendaele, M., & Van Damme, I. (2018). Reduced contamination of pig carcasses using an alternative pluck set removal procedure during slaughter. *Meat science*, 145, 23-30.

Van Ba, H., Seo, H. W., Seong, P. N., Kang, S. M., Cho, S. H., Kim, Y. S., ... & Kim, J. H. (2019). The fates of microbial populations on pig carcasses during slaughtering process, on retail cuts after slaughter, and intervention efficiency of lactic acid spraying. *International journal of food microbiology*, 294, 10-17.

Koch, F., Wiacek, C., & Braun, P. G. (2019). Pulsed light treatment for the reduction of *Salmonella* Typhimurium and *Yersinia enterocolitica* on pork skin and pork loin. *International journal of food microbiology*, 292, 64-71.

Nesbakken, T., Eckner, K., & Røtterud, O. J. (2008). The effect of blast chilling on occurrence of human pathogenic *Yersinia enterocolitica* compared to *Campylobacter* spp. and numbers of hygienic indicators on pig carcasses. *International journal of food microbiology*, 123(1-2), 130-133.

Zhou, Z., Jin, X., Zheng, H., Li, J., Meng, C., Yin, K., Xie, X., Huang, C., Lei, T., Sun, X., Xia, Z., Zeng, Y., Pan, Z., Jiao, X., 2018. The prevalence and load of *Salmonella*, and key risk points of *Salmonella* contamination in a swine slaughterhouse in Jiangsu province, China. *Food Control* 87, 153-160.

**Relevance confirmation form**

| Question | Options | Notes |
|---|---|---|
| **Relevance confirmation** | | |
| Does this article investigate primary research on the efficacy and/or effectiveness of interventions to control microbiological contamination (with indicator bacteria and pathogens) in pork at any stage in production chain from pigs received in abattoir to the carcass chilling step inclusive (abattoir level) and meet the PICOS eligibility criteria? | ☐ Yes (proceed further)<br>☐ No (summarise it narratively)<br>- previous systematic reviews, risk assessments and stochastic models, cost-benefit analysis<br>☐ No (exclude)<br>– measures irrelevant population (species other than pigs; pork meat after carcass chilling (fabricated, cured, fermented, dried, tenderised, marinated and ready-to-eat meat); environment (surfaces, equipment, scald water, etc)<br>– measures irrelevant outcome (i.e. spoilage)<br>– in vitro study (model broth systems)<br>– not primary research<br>– not retrievable<br>– duplicate data, specify: ________<br>– no intervention measured<br>– other language<br>– other, specify: _______________ | ***Primary research** is defined as original research during which authors generated and reported their own data<br><br>*"**PICOS**" elements summarise the population (P), the intervention (I), the comparator (C), the main outcome (O) and the study design chosen (S)<br><br>***Interventions**: pigs handling in lairage, logistic slaughter, standard practices (scalding, singeing, evisceration, dressing), carcass decontamination (pre-chill), chilling (dry, spray, blast), multiple interventions |

| Key primary research article characteristics (record only for included articles) | | |
|---|---|---|
| What type of document is this article? | ☐ Journal article<br>☐ Conference paper<br>☐ Government or research report<br>☐ Thesis<br>☐ Book or book chapter<br>☐ Other, specify ______________ | |
| In what regions and country was the study conducted? | ☐ North America: _______________<br>☐ Europe: _____________________<br>☐ Australasia: __________________<br>☐ Central and South America/ Caribbean: ____________________<br>☐ Asia: ______________________<br>☐ Africa: _____________________<br>☐ Not stated: __________________ | |
| Study design: | ☐ Experimental research:<br>– Controlled trial<br>– Challenge trial<br>– Before-and-after trial<br>☐ Observational research<br>– Cohort study<br>– Case-control study<br>– Cross-sectional study<br>– Other: _____________________ | |
| In what setting was the study carried out? | ☐ Commercial/abattoir conditions<br>☐ Research/pilot plant<br>☐ Laboratory conditions<br>☐ Not reported | **Pilot plant**: Experiments using industrial equipment in non-industrial settings |
| What stage in the pork production chain and category of intervention(s) are investigated in this article? | ☐ Abattoir (pre-slaughter, lairage):<br>– Pigs handling in lairage<br>– Logistic slaughter<br>☐ Abattoir processing:<br>– Scalding<br>– Singeing | **Multiple interventions** (multiple-hurdle strategy): usually interventions placed in a single step or |

| | | |
|---|---|---|
| | – Standard processing procedures/GHP<br>– Carcass interventions (pre- and post-evisceration, pre-chill)<br>– Chilling, spray chilling, blast chilling<br>– Multiple interventions | (more often) in consecutive steps on a processing line |
| Specify intervention(s) | – ______________________ | |
| What outcomes did the study investigate? | ☐ Aerobic colony counts<br>☐ *Enterobacteriaceae* counts<br>☐ Generic *E. coli* counts<br>☐ *Salmonella*<br>☐ *Yersinia enterocolitica*<br>☐ *Yersinia pseudotuberculosis* | |
| What outcome measures are investigated? | ☐ Efficacy<br>☐ Cost/practicality<br>☐ Consumer acceptability | |
| Does the article report any extractable data about pig interventions' effectiveness that could be used for possible meta-analysis? | ☐ Yes, proceed to Risk-of-bias assessment<br>☐ No, proceed to Data extraction and summarise it narratively.<br>– no measure of variability<br>☐ No, use it for contextual purposes. Specify reason:<br>– graphical data only<br>– other: ______________________ | *Extractable data examples: prevalence; concentration; OR, RR<br><br>*No extractable data if there is no data reported about:<br>- number of samples;<br>- standard deviation, standard error or confidence intervals;<br>- mean treatment and control |

**Risk of bias form**

| Bias domain | Signaling questions |
|---|---|
| Bias arising from the randomization process | 1.1. Was the allocation sequence random? |
| | 1.2. Was the allocation sequence concealed until samples were assigned to interventions? |
| | 1.3. Did baseline differences between groups suggest a problem with the randomization process? |
| Bias due to deviations from the intended interventions | 2.1. Were researchers aware of the assigned interventions? |
| | 2.2. Were there deviations from the planned interventions/ methodologies? |
| | 2.3. Did these deviations likely affect the outcome? |
| | 2.4. Are there any concerns that confounders have not been appropriately identified and accounted for? |
| Bias due to missing outcome data | 3.1. Were there missing data? |
| | 3.2. Would the level of missing data affect the outcome data? |
| Bias in measurement of the outcome | 4.1. Was the method of measuring the outcome appropriate? |
| | 4.2. Could measurement of the outcome differ between groups? |
| | 4.3. Were outcome assessors aware of the intervention groups? |
| | 4.4. Could assessment of outcome be influenced by knowledge of intervention received? |
| | 4.5. Is it likely that assessment of outcome was influenced by knowledge of intervention? |
| Bias in selection of the reported result | 5.1. Did authors report all outcomes? |
| | 5.2. Did the outcomes match with the intended aim and plan of the study? |
| | 5.3. Is there an appropriate justification why the outcome measure was selected? |

**Data extraction form**

| Question | Options |
| --- | --- |
| Specify intervention stage in the pork production chain where intervention is applied | 1. Abattoir pre-slaughter (lairage interventions)<br>2. Abattoir processing<br>– Pre-evisceration<br>– Post-evisceration, pre-chill<br>– Chilling |
| Specify broad intervention category (and subcategory) being extracted | – Pigs handling in lairage<br>– Logistic slaughter<br>– Standard processing procedures/GHP<br>– Carcass interventions (pre-chill)<br>– Chilling, spray chilling, blast chilling<br>– Multiple interventions |
| Specify intervention | – ________________________ |
| Intervention description (concentration, temperature, application method, contact time, pressure) | – ________________________ |
| Specify target (intervention) population/sample category to which intervention is applied | 1. Live animal<br>2. Carcass surface |
| Specify target (intervention) population/sample more in details | – ________________________ |
| Specify outcome sample category | 1. Live animal<br>2. Carcass surface |
| What type of outcome sample was measured? | 1. Swab (sponge, other)<br>2. Excised meat sample |
| Specify comparison (control) group | 1. No treatment<br>2. Pre treatment<br>3. Water wash<br>4. Other: ____________________ |
| What outcome group did the study investigate? | 1. Aerobic colony counts (ACC)<br>2. *Enterobacteriaceae* counts (EBC)<br>3. Generic *E. coli* counts<br>4. *Salmonella*<br>5. *Yersinia enterocolitica*<br>6. *Yersinia pseudotuberculosis* |
| What outcome strains did the study | – ________________________ |

| | |
|---|---|
| investigate? | |
| What outcome data were measured? | 1. Concentration (log CFU): specify area of measurement: _______________<br>2. Prevalence (presence/absence) |
| Extract quantitative outcome data in text boxes for each relevant Category | **Concentration outcomes**<br>– Mean of control (Mc)<br>– Standard deviation of control group (CDc)<br>– Standard error of control group (SEMc)<br>– Confidence interval of control group (CIc)<br>– Number in control group (Nc)<br>– Mean of intervention group (Me)<br>– Standard deviation of intervention group (SDe)<br>– Standard error of intervention group (SEMe)<br>– Confidence interval of intervention group (CIe)<br>– Number in intervention group (Ne)<br><br>**Prevalence outcomes**<br>– Number of events (i.e. positives) in control group (Ec)<br>– Number of participants in control group (Nc)<br>– Number of events (i.e. positives) in intervention group (Ee)<br>– Number of participants in intervention group (Ne) |

**References for studies used in meta-analysis**


1. Biasino, W., De Zutter, L., Woollard, J., Mattheus, W., Bertrand, S., Uyttendaele, M., Van Damme, I., 2018. Reduced contamination of pig carcasses using an alternative pluck set removal procedure during slaughter. *Meat Science* 145, 23-30.
2. Chang, V.P., Mills, E.W., Cutter, C.N., 2003. Reduction of bacteria on pork carcasses associated with chilling method. *Journal of Food Protection* 66, 1019-1024.
3. Gill, C., Bedard, D., Jones, T., 1997. The decontaminating performance of a commercial apparatus for pasteurizing polished pig carcasses. *Food Microbiology* 14, 71-79.
4. Gill, C.O., Dussault, F., Holley, R.A., Houde, A., Jones, T., Rheault, N., Rosales, A., Quessy, S., 2000. Evaluation of the hygienic performances of the processes for cleaning, dressing and cooling pig carcasses at eight packing plants. *International Journal of Food Microbiology* 58, 65-72.
5. Gill, C.O., Jones, T., 1997. Assessment of the hygienic performances of an air-cooling process for lamb carcasses and a spray-cooling process for pig carcasses. *International Journal of Food Microbiology* 38, 85-93.
6. Gill, C.O., Jones, T., Badoni, M., 1998. The effects of hot water pasteurizing treatments on the microbiological conditions and appearances of pig and sheep carcasses. *Food Research International* 31, 273-278.
7. Hamilton, D., Holds, G., Lorimer, M., Kiermeier, A., Kidd, C., Slade, J., Pointon, A., 2010. Slaughterfloor decontamination of pork carcases with hot water or acidified sodium chlorite - A comparison in two Australian abattoirs. *Zoonoses and Public Health* 57, 16-22.
8. Koch, F., Wiacek, C., Braun, P.G., 2019. Pulsed light treatment for the reduction of *Salmonella* Typhimurium and *Yersinia enterocolitica* on pork skin and pork loin. *International Journal of Food Microbiology* 292, 64-71.
9. Langkabel, N., Großpietsch, R., Oetjen, M., Bräutigam, L., Irsigler, H., Jaeger, D., Ludewig, R., Fries, R., 2014. Microbiological status of pig carcasses in mobile chilling vehicles. *Archiv fur Lebensmittelhygiene* 65, 45-49.
10. Laukkanen, R., Ranta, J., Dong, X., Hakkinen, M., Martínez, P.O., Lundén, J., Johansson, T., Korkeala, H., 2010. Reduction of enteropathogenic *Yersinia* in the pig slaughterhouse by using bagging of the rectum. *Journal of Food Protection* 73, 2161-2168.
11. Martín-Peláez, S., Martín-Orúe, S.M., Pérez, J.F., Fàbrega, E., Tibau, J., Gasa, J., 2008. Increasing feed withdrawal and lairage times prior to slaughter decreases the gastrointestinal tract weight but favours the growth of cecal *Enterobacteriaceae* in pigs. *Livestock Science* 119, 70-76.
12. Nesbakken, T., Nerbrink, E., Røtterud, O.J., Borch, E., 1994. Reduction of *Yersinia enterocolitica* and *Listeria* spp. on pig carcasses by enclosure of the rectum during slaughter. *International Journal of Food Microbiology* 23, 197-208.
13. Pearce, R.A., Bolton, D.J., Sheridan, J.J., McDowell, D.A., Blair, I.S., Harrington, D., 2004. Studies to determine the critical control points in pork slaughter hazard analysis and critical control point systems. *International Journal of Food Microbiology* 90, 331-339.
14. Purnell, G., James, C., Wilkin, C.A., James, S.J., 2010. An evaluation of improvements in carcass hygiene through the use of anal plugging of pig carcasses prior to scalding and dehairing. *Journal of Food Protection* 73, 1108-1110.
15. Rahkio, M., Korkeala, H., Sippola, I., Peltonen, M., 1992. Effect of pre-scalding brushing on contamination level of pork carcasses during the slaughtering process. *Meat Science* 32, 173-183.
16. Rivas, T., Vizcaíno, J.A., Herrera, F.J., 2000. Microbial contamination of carcasses and equipment from an Iberian pig slaughterhouse. *Journal of Food Protection* 63, 1670-1675.
17. Spescha, C., Stephan, R., Zweifel, C., 2006. Microbiological contamination of pig carcases at different stages of slaughter in two Europian Union-approved abattoirs. *Journal of Food Protection* 69, 2568-2575.

18. Van Ba, H., Seo, H.W., Seong, P.N., Kang, S.M., Cho, S.H., Kim, Y.S., Park, B.Y., Moon, S.S., Kang, S.J., Choi, Y.M., Kim, J.H., 2019. The fates of microbial populations on pig carcasses during slaughtering process, on retail cuts after slaughter, and intervention efficiency of lactic acid spraying. *International Journal of Food Microbiology* 294, 10-17.
19. Van Netten, P., Mossel, D.A.A., Huis In 'T Veld, J.H.J., 1997. Microbial changes on freshly slaughtered pork carcasses due to "hot" lactic acid decontamination. *Journal of Food Safety* 17, 89-111.
20. Van Netten, P., Valentijn, A., Mossel, D.A.A., Huis In 'T Veld, J.H.J., 1997. Fate of low temperature and acid-adapted *Yersinia enterocolitica* and *Listeria monocytogenes* that contaminate lactic acid decontaminated meat during chill storage. *Journal of Applied Microbiology* 82, 769-779.
21. Walia, K., Lynch, H., Grant, J., Duffy, G., Leonard, F.C., Lawlor, P.G., Gardiner, G.E., 2017. The efficacy of disinfectant misting in the lairage of a pig abattoir to reduce *Salmonella* and *Enterobacteriaceae* on pigs prior to slaughter. *Food Control* 75, 55-61.
22. Yu, S., Bolton, D., Laubach, C., Kline, P., Oser, A., Palumbo, S.A., 1999. Effect of dehairing operations on microbiological quality of swine carcasses. *Journal of Food Protection* 62, 1478-1481.

**Efficacies of pig carcass interventions from studies where there was only one trial reported (comparison group was available)**

| **Intervention** | **Microorganism** [a] | **Study design/ conditions**‡ | **Description** | **$Log_{10}$ mean difference (95%-CI)** | **Study** |
|---|---|---|---|---|---|
| Water misting live pigs | EBC | CT/comm | 30 mins | 0.61 (-0.94 - 2.16) | Walia et al., (2017) |
| Disinfectant Virkon® S misting vs water misting live pigs | EBC | CT/comm | 30 mins | -1.36 (-2.91 - 0.19) | |
| Disinfectant Virkon® S misting live pigs | EBC | CT/comm | 30 mins | -0.75 (-2.3 - 0.8) | |
| Carcass water wash | *E. coli* | CT/comm | 25 s, high pressure | -0.11 (-0.27 - 0.05) | Rivas et al., (2000) |
| Carcass water wash | EBC | CT/comm | 25 s, high pressure | -0.11 (-0.27 - 0.05) | |
| Anal plugging | EBC | CT/comm | Two plugs before scalding and dehairing | -1.10 (-1.58 - -0.62) | Purnell et al., (2010) |
| Blast and water spray carcass chilling | *E. coli* | BA/comm | Blast at -20°C for 1h, then water at 5°C for 20s, 10 min intervals | -0.03 (-0.37 - 0.31) | Gill and Jones (1997) |
| Dehairing | *E. coli* | BA/comm | Dehairing | 0.35 (0.29-0.41) | Rivas et al., (2000) |
| Scalding | *E. coli* | BA/comm | Scalding | -3.26 (-3.32 - -3.2) | |
| Scalding | EBC | BA/comm | Scalding | -3.42 (-3.47 - -3.37) | |
| Dehairing | EBC | BA/comm | Dehairing | 0.72 (0.65 - 0.79) | |
| Multiple interventions | EBC | BA/comm | Scalding, dehairing, singeing, scraping | -2.15 (-2.28 - -2.02) | |
| Multiple interventions | *E. coli* | BA/comm | Scalding, dehairing, singeing, scraping | -2.20 (-2.34 - -2.06) | |
| Multiple interventions | ACC | BA/comm | Scalding, dehairing, singeing, scraping | -0.87 (-0.91 - -0.83) | |

‡ CT-controlled trial; BA-before-and-after trial; Comm-commercial abattoir conditions.

[a] ACC-aerobic colony count; EBC-*Enterobacteriaceae* count

**Examples of intervention forest plots**

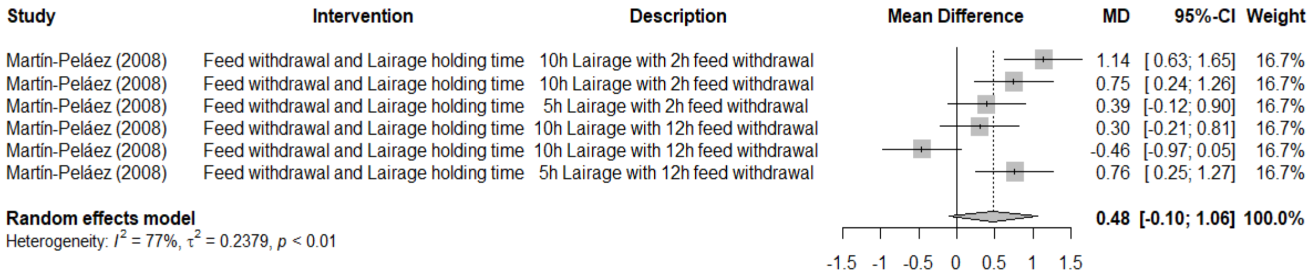


**Supplementary Figure S1.** Forest plot of the results of cohort trials performed under experimental farm conditions to investigate the efficacy of feed withdrawal and lairage holding time compared to control group with less lairage time and feed withdrawal period in reducing *Enterobacteriaceae* count (log 16S rRNA gene copies) in pig caecal content.

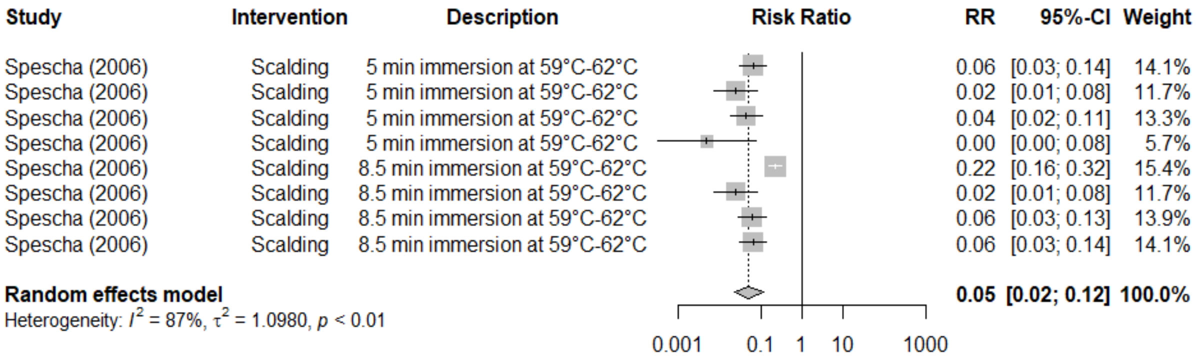


**Supplementary Figure S2.** Forest plot of the results of before-and-after trials performed under commercial abattoir conditions to investigate the efficacy of scalding in reducing *Enterobacteriaceae* prevalence on pig carcasses.

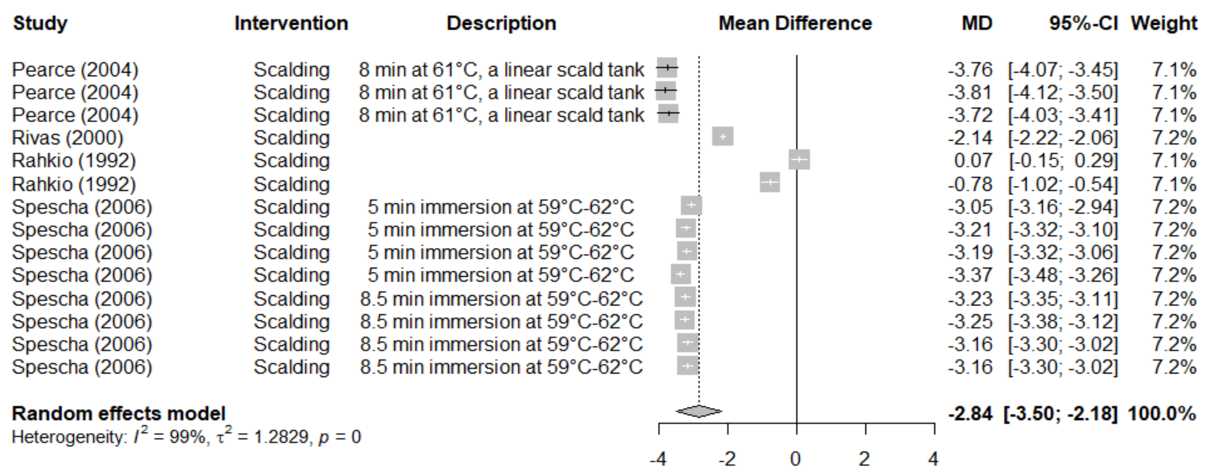


**Supplementary Figure S3.** Forest plot of the results of before-and-after trials performed under commercial abattoir conditions to investigate the efficacy of scalding in reducing aerobic colony count ($log_{10}$ CFU) on pig carcasses.

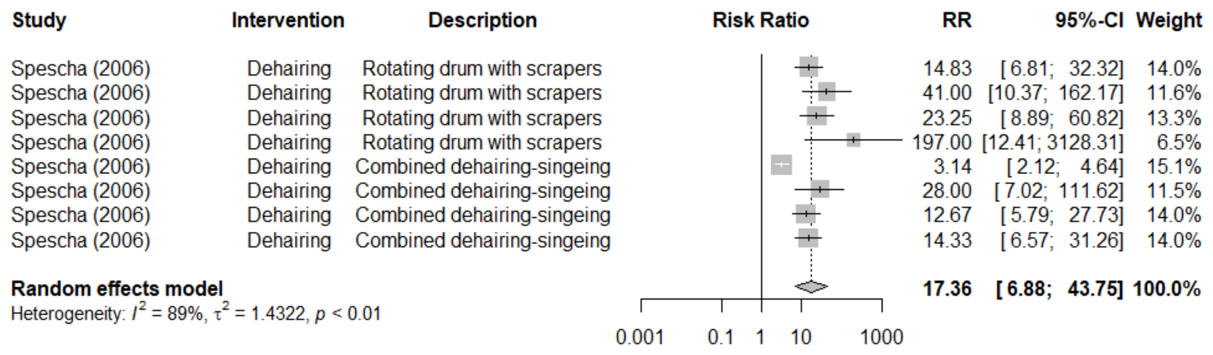


**Supplementary Figure S4**. Forest plot of the results of before-and-after trials performed under commercial abattoir conditions to investigate the efficacy of dehairing in reducing *Enterobacteriaceae* prevalence on pig carcasses.

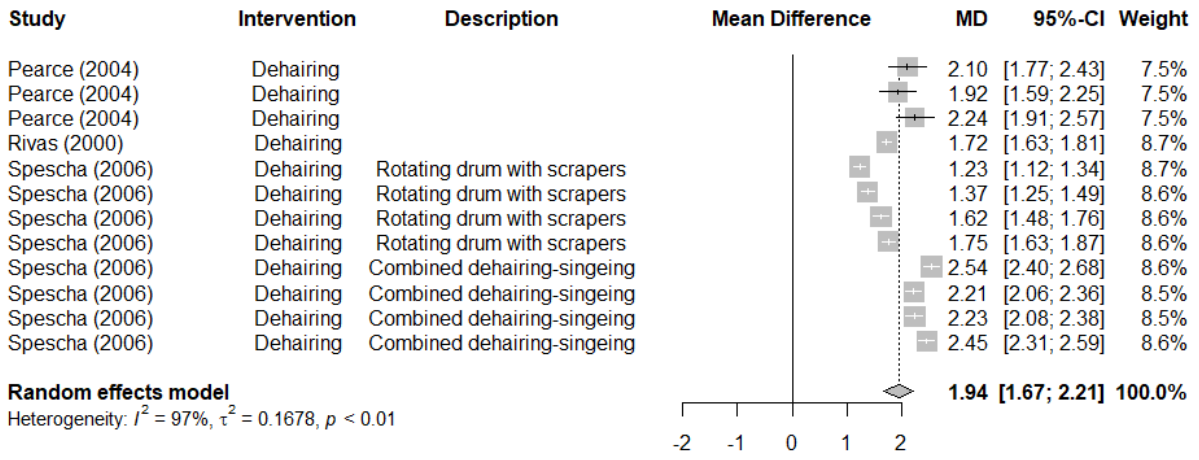


**Supplementary Figure S5**. Forest plot of the results of before-and-after trials performed under commercial abattoir conditions to investigate the efficacy of dehairing in reducing aerobic colony count ($log_{10}$ CFU) on pig carcasses.

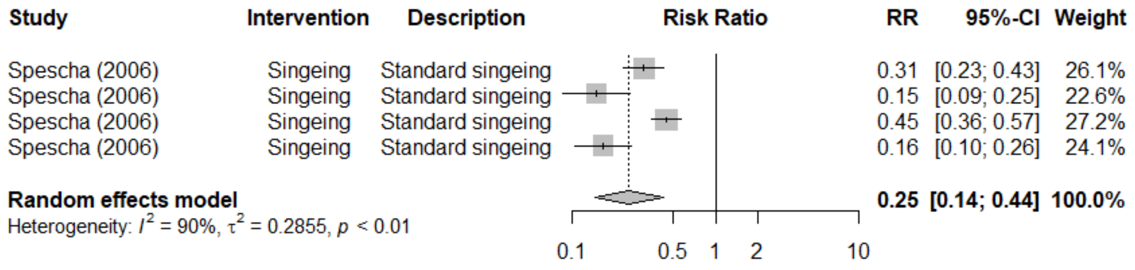


**Supplementary Figure S6.** Forest plot of the results of before-and-after trials performed under commercial abattoir conditions to investigate the efficacy of singeing in reducing *Enterobacteriaceae* prevalence on pig carcasses.

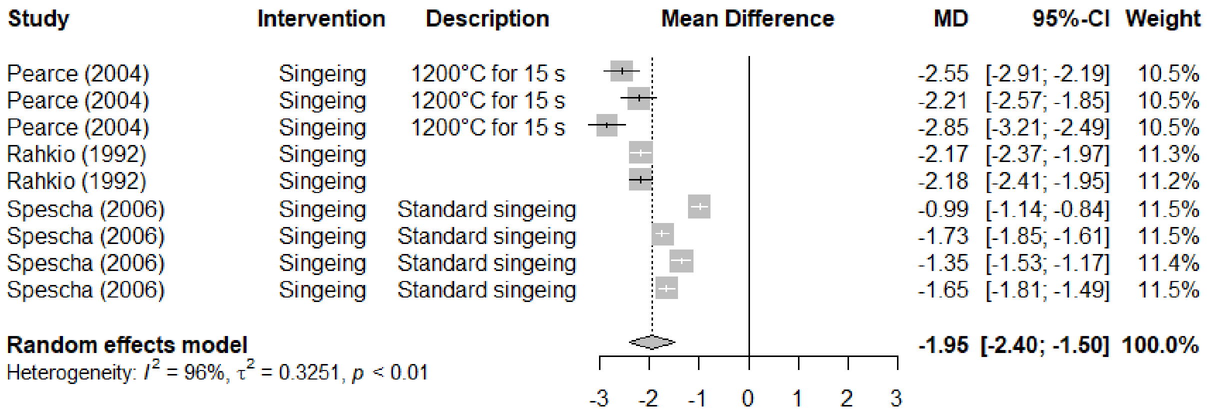


**Supplementary Figure S7.** Forest plot of the results of before-and-after trials performed under commercial abattoir conditions to investigate the efficacy of singeing in reducing aerobic colony count ($log_{10}$ CFU) on pig carcasses.

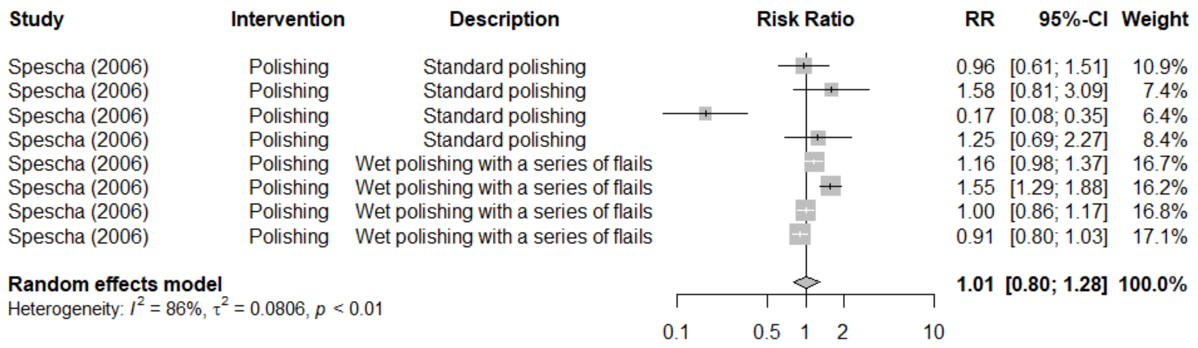


**Supplementary Figure S8**. Forest plot of the results of before-and-after trials performed under commercial abattoir conditions to investigate the efficacy of polishing in reducing *Enterobacteriaceae* prevalence on pig carcasses.

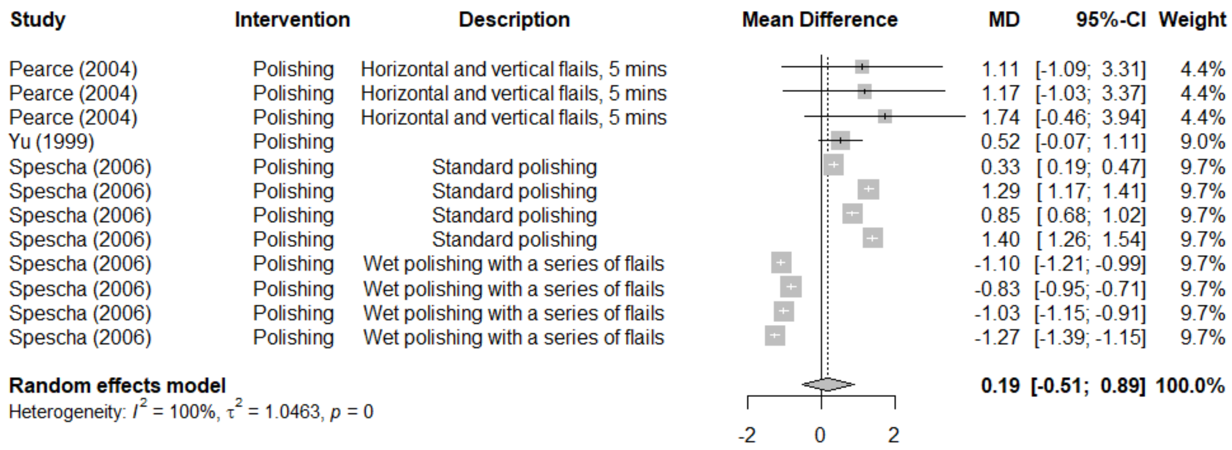


**Supplementary Figure S9**. Forest plot of the results of before-and-after trials performed under commercial abattoir conditions to investigate the efficacy of polishing in reducing aerobic colony count ($log_{10}$ CFU) on pig carcasses.

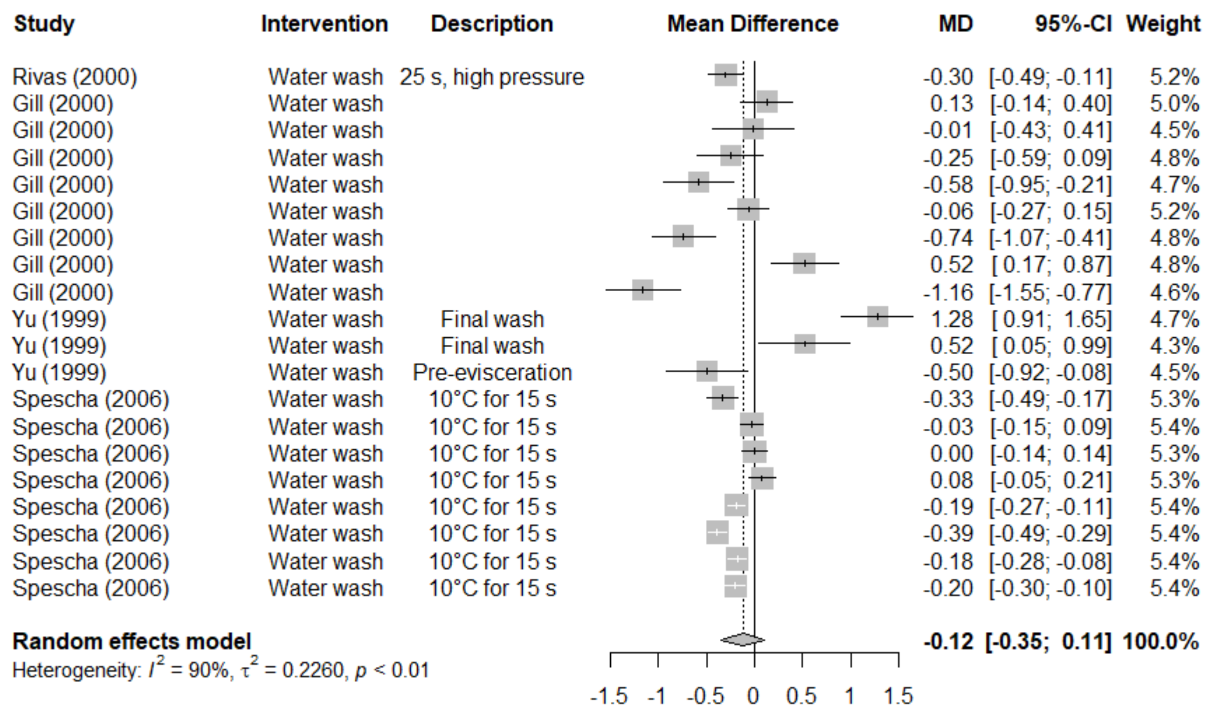


**Supplementary Figure S10**. Forest plot of the results of combined controlled trials and before-and-after trials performed under commercial abattoir conditions to investigate the efficacy of water washing in reducing aerobic colony count ($log_{10}$ CFU) on pig carcasses.

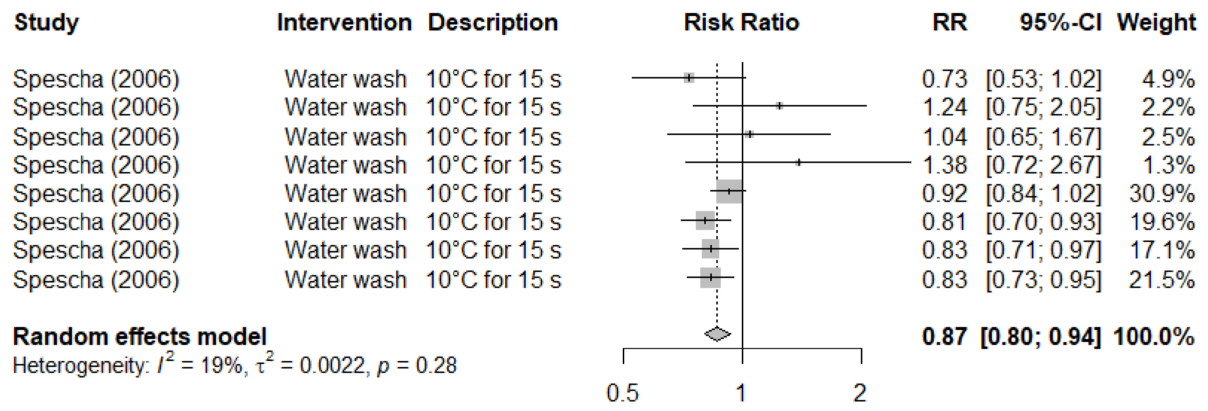


**Supplementary Figure S11.** Forest plot of the results of before-and-after trials performed under commercial abattoir conditions to investigate the efficacy of water washing in reducing *Enterobacteriaceae* prevalence on pig carcasses.

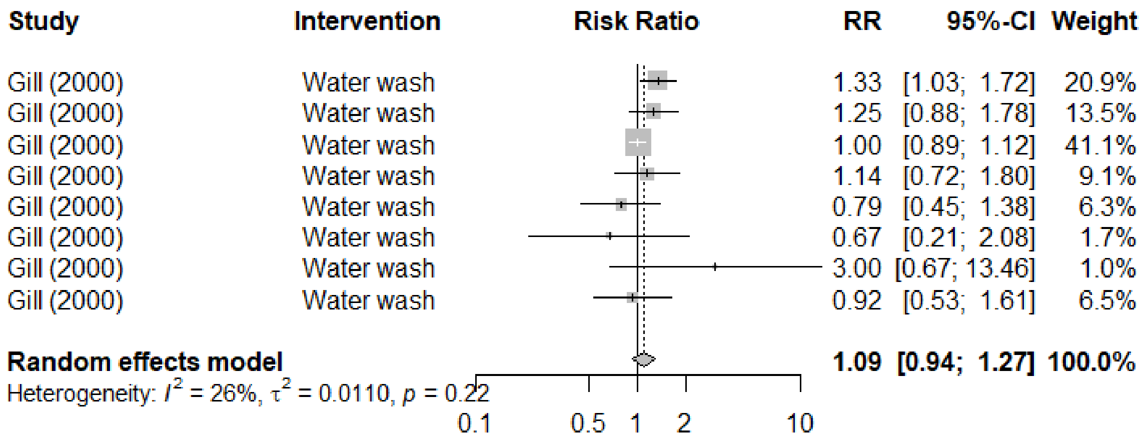


**Supplementary Figure S12.** Forest plot of the results of before-and-after trials performed under commercial abattoir conditions to investigate the efficacy of water washing in reducing generic *E. coli* prevalence on pig carcasses.

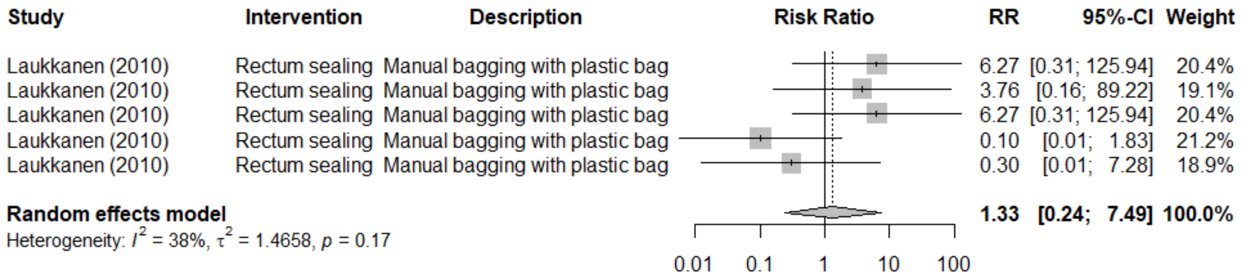


**Supplementary Figure S13.** Forest plot of the results of controlled trials performed under commercial abattoir conditions to investigate the efficacy of rectum sealing in reducing *Yersinia pseudotuberculosis* prevalence on pig carcasses.

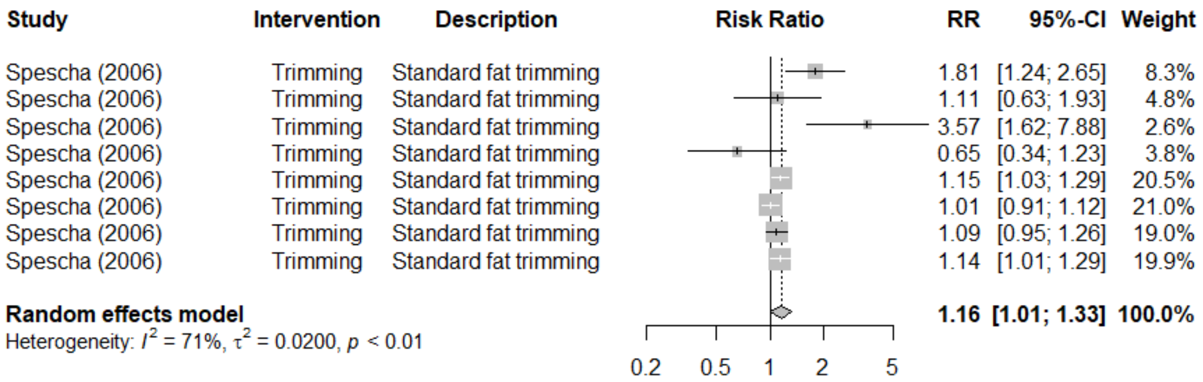


**Supplementary Figure S14.** Forest plot of the results of before-and-after trials performed under commercial abattoir conditions to investigate the efficacy of trimming in reducing *Enterobacteriaceae* prevalence on pig carcasses.

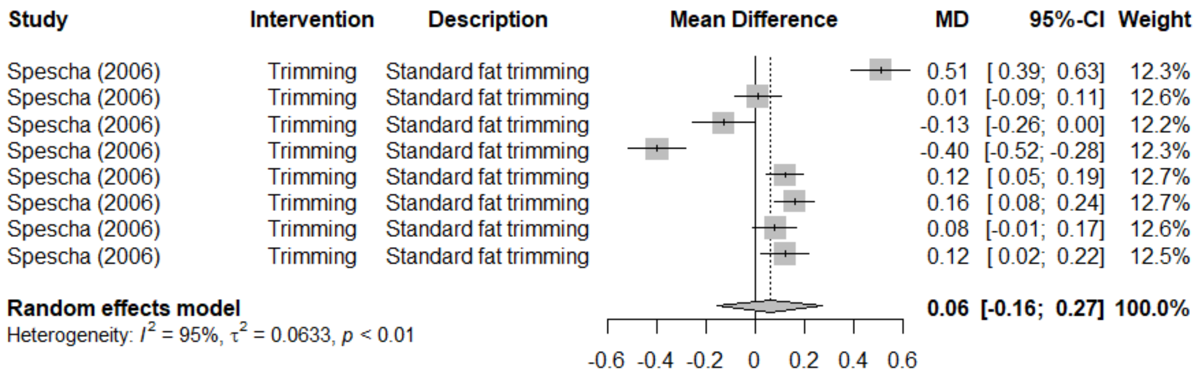


**Supplementary Figure S15.** Forest plot of the results of before-and-after trials performed under commercial abattoir conditions to investigate the efficacy of trimming in reducing aerobic colony count ($log_{10}$ CFU) on pig carcasses.

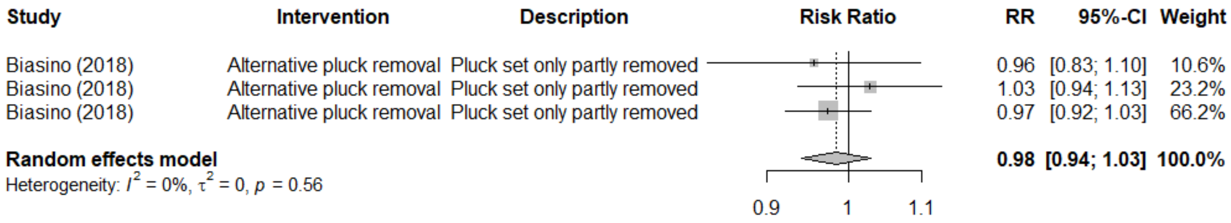


**Supplementary Figure S16.** Forest plot of the results of controlled trials performed under commercial abattoir conditions to investigate the efficacy of alternative pluck removal compared to standard pluck removal in reducing *Enterobacteriaceae* prevalence on pig carcasses.

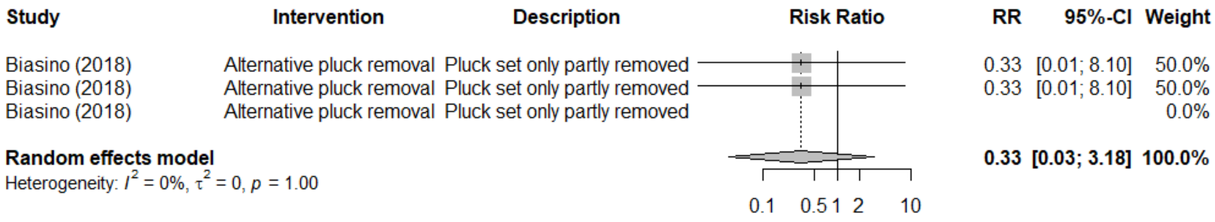


**Supplementary Figure S17.** Forest plot of the results of controlled trials performed under commercial abattoir conditions to investigate the efficacy of alternative pluck removal compared to standard pluck removal in reducing *Yersinia enterocolitica* prevalence on pig carcasses.

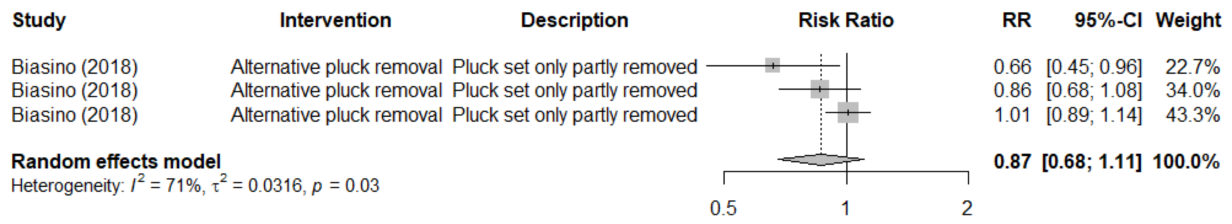


**Supplementary Figure S18.** Forest plot of the results of controlled trials performed under commercial abattoir conditions to investigate the efficacy of alternative pluck removal compared to standard pluck removal in reducing generic *E. coli* prevalence on pig carcasses.

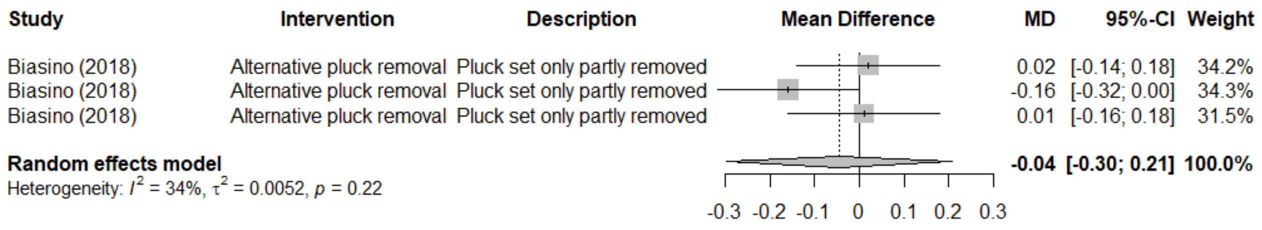


**Supplementary Figure S19**. Forest plot of the results of controlled trials performed under commercial abattoir conditions to investigate the efficacy of alternative pluck removal compared to standard pluck removal in reducing aerobic colony count ($log_{10}$ CFU) transfer on pig carcasses.

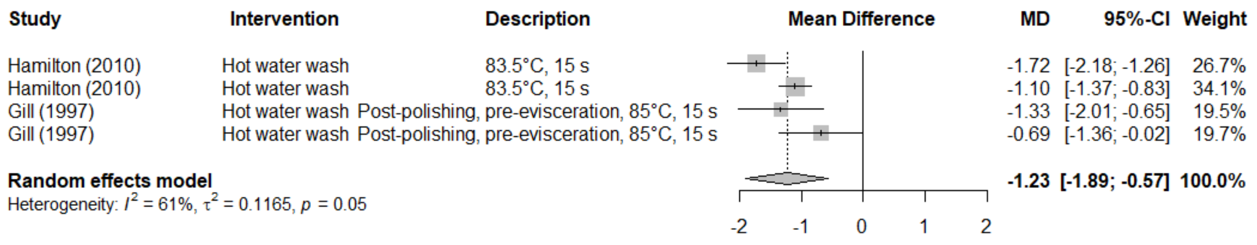


**Supplementary Figure S20**. Forest plot of the results of combined controlled trials and before-and-after trials performed under commercial abattoir conditions to investigate the efficacy of hot water washing in reducing generic *E. coli* count ($log_{10}$ CFU) on pig carcasses.

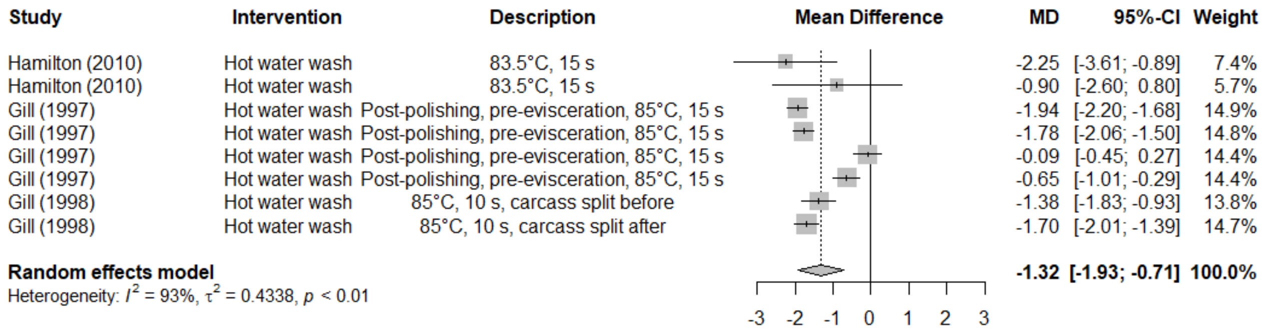


**Supplementary Figure S21.** Forest plot of the results of combined controlled trials and before-and-after trials performed under commercial abattoir conditions to investigate the efficacy of hot water washing in reducing aerobic colony count ($log_{10}$ CFU) on pig carcasses.

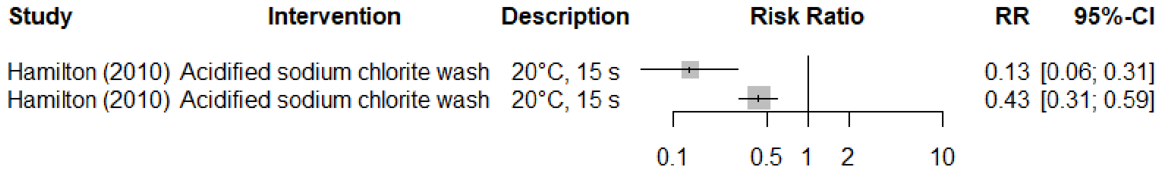


**Supplementary Figure S22**. Forest plot of the results of controlled trials performed under commercial abattoir conditions to investigate the efficacy of acidified sodium chlorite washing in reducing generic *E. coli* prevalence on pig carcasses.

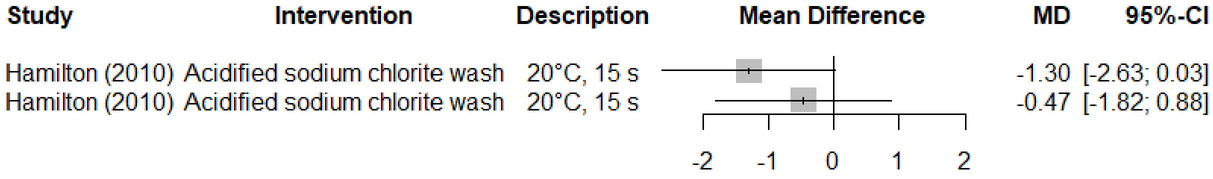


**Supplementary Figure S23**. Forest plot of the results of controlled trials performed under commercial abattoir conditions to investigate the efficacy of acidified sodium chlorite washing in reducing aerobic colony count ($log_{10}$ CFU) on pig carcasses.

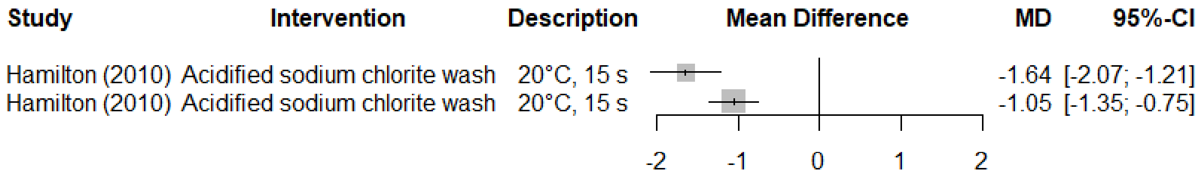


**Supplementary Figure S24**. Forest plot of the results of controlled trials performed under commercial abattoir conditions to investigate the efficacy of acidified sodium chlorite washing in reducing generic *E. coli* count ($\log_{10}$ CFU) on pig carcasses.

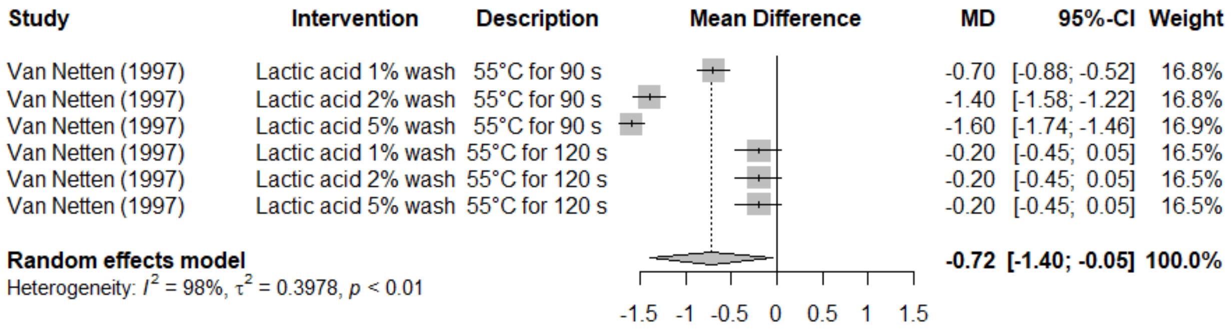


**Supplementary Figure S25**. Forest plot of the results of challenge trials performed under laboratory conditions to investigate the efficacy of lactic acid washing in reducing *Enterobacteriaceae* count ($\log_{10}$ CFU) on pig carcass meat.

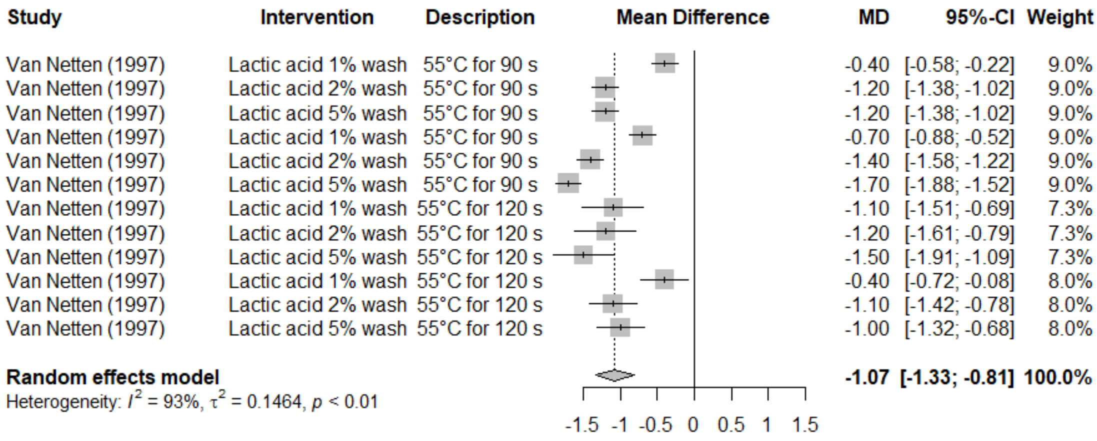


**Supplementary Figure S26**. Forest plot of the results of challenge trials performed under laboratory conditions to investigate the efficacy of lactic acid washing in reducing aerobic colony count ($\log_{10}$ CFU) on pig carcass meat.

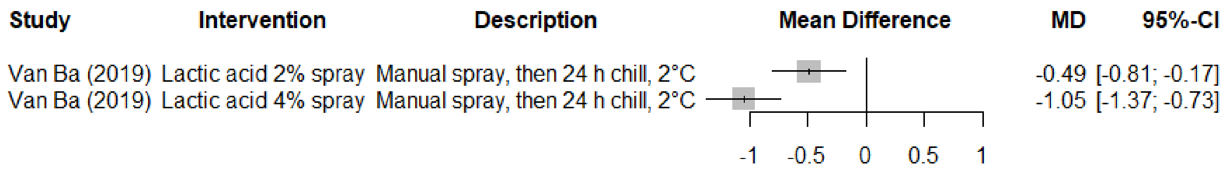


**Supplementary Figure S27**. Forest plot of the results of controlled trials performed under commercial abattoir conditions to investigate the efficacy of lactic acid washing in reducing aerobic colony count ($log_{10}$ CFU) on pig carcasses.

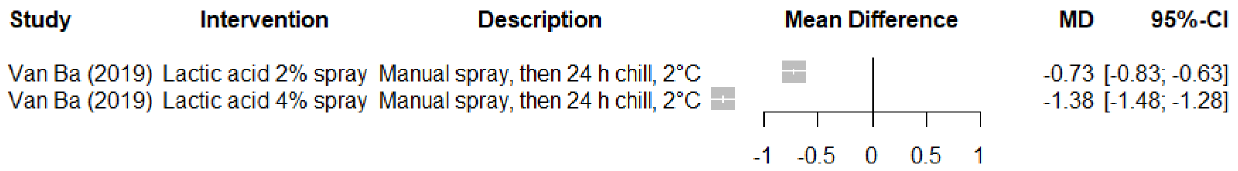


**Supplementary Figure S28**. Forest plot of the results of controlled trials performed under commercial abattoir conditions to investigate the efficacy of lactic acid washing in reducing generic *E. coli* count ($log_{10}$ CFU) on pig carcasses.

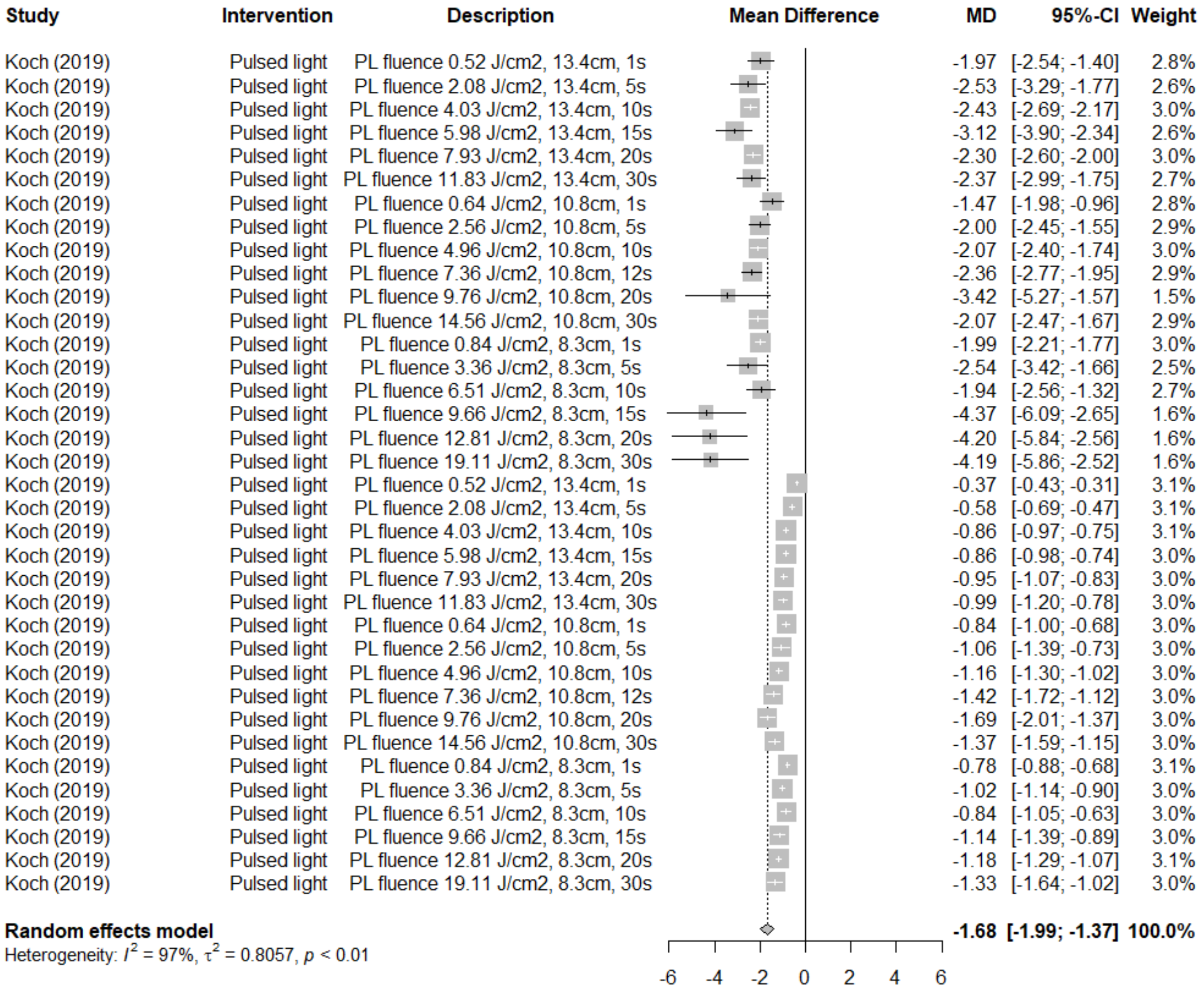


**Supplementary Figure S29**. Forest plot of the results of challenge trials performed under laboratory conditions to investigate the efficacy of pulsed light treatment in reducing *Yersinia enterocolitica* counts ($log_{10}$ CFU) on pig carcass meat.

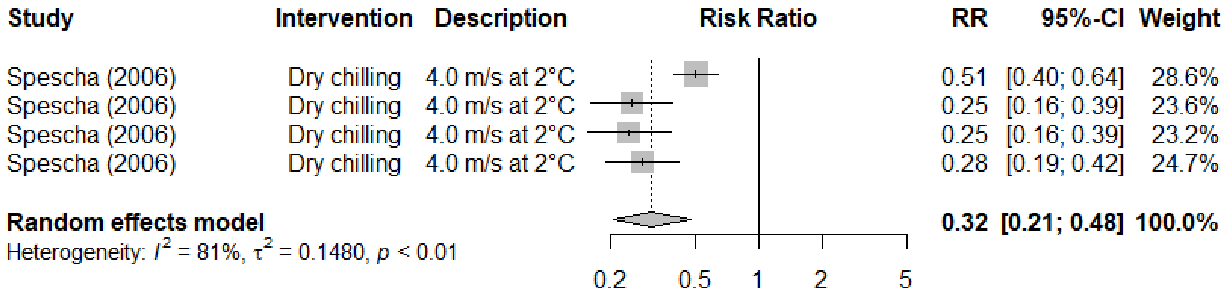


**Supplementary Figure S30**. Forest plot of the results of before-and-after trials performed under commercial abattoir conditions to investigate the efficacy of dry chilling in reducing *Enterobacteriaceae* prevalence on pig carcasses.

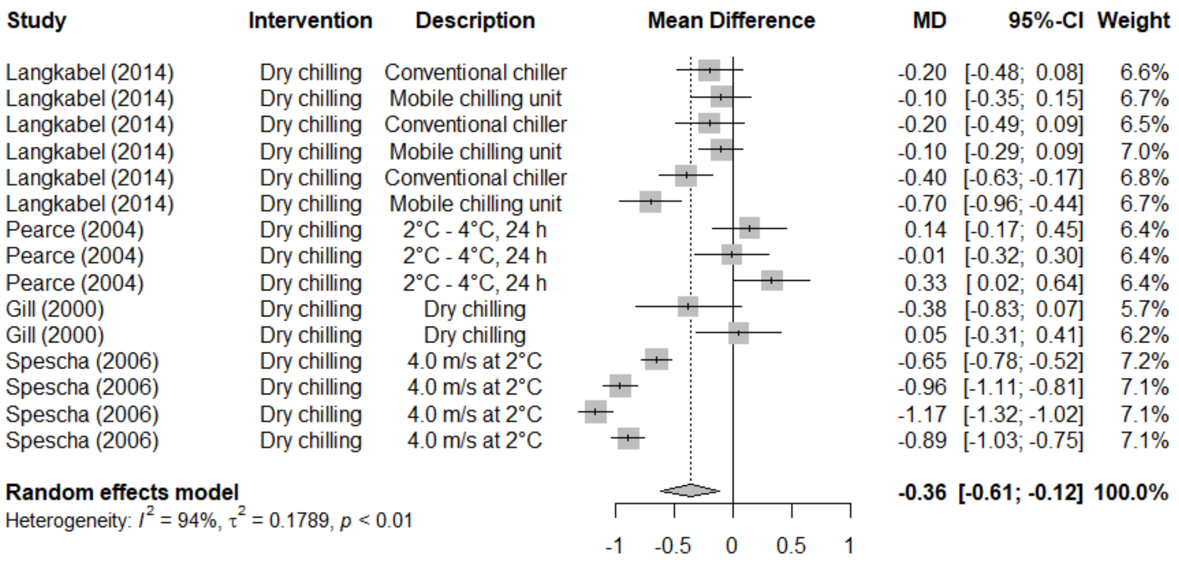


**Supplementary Figure S31**. Forest plot of the results of before-and-after trials performed under commercial abattoir conditions to investigate the efficacy of dry chilling in reducing aerobic colony count ($log_{10}$ CFU) on pig carcasses.

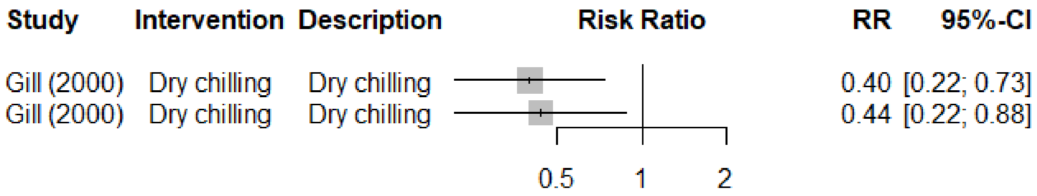


**Supplementary Figure S32**. Forest plot of the results of before-and-after trials performed under commercial abattoir conditions to investigate the efficacy of dry chilling in reducing generic *E. coli* prevalence on pig carcasses.

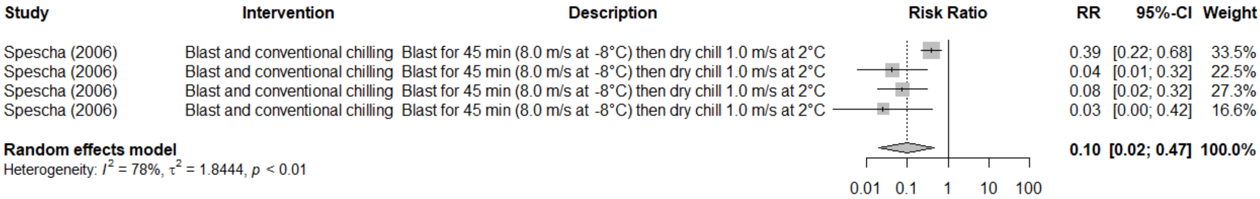


**Supplementary Figure S33**. Forest plot of the results of before-and-after trials performed under commercial abattoir conditions to investigate the efficacy of blast and conventional chilling in reducing *Enterobacteriaceae* prevalence on pig carcasses.

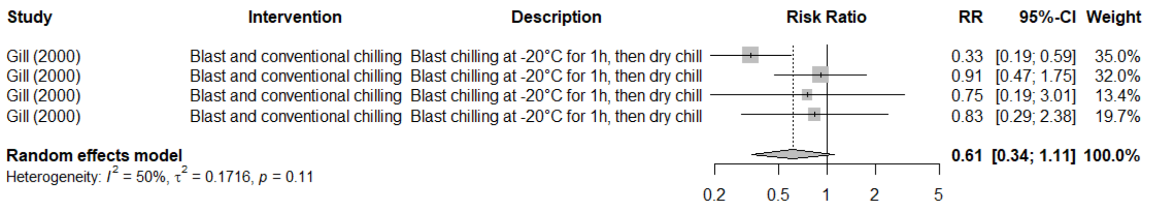


**Supplementary Figure S34**. Forest plot of the results of before-and-after trials performed under commercial abattoir conditions to investigate the efficacy of blast and conventional chilling in reducing generic *E. coli* prevalence on pig carcasses.

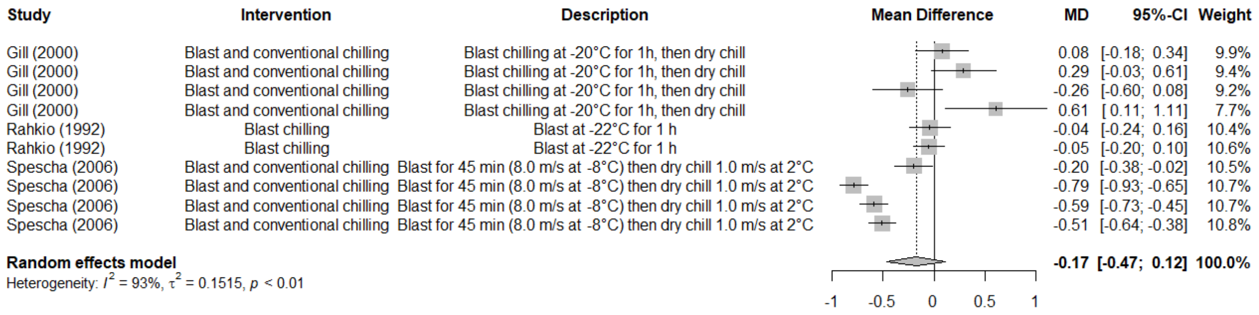


**Supplementary Figure S35**. Forest plot of the results of before-and-after trials performed under commercial abattoir conditions to investigate the efficacy of blast and conventional chilling in reducing aerobic colony count ($log_{10}$ CFU) on pig carcasses.

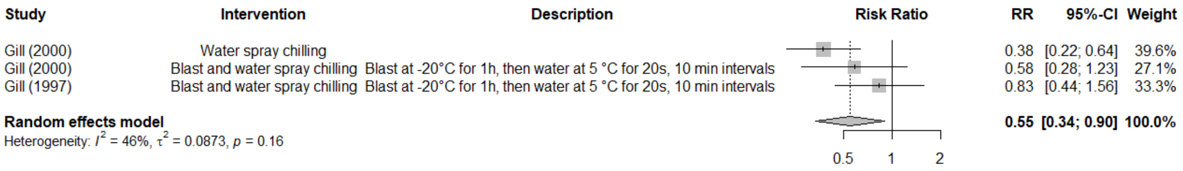


**Supplementary Figure S36**. Forest plot of the results of before-and-after trials performed under commercial abattoir conditions to investigate the efficacy of blast and water spray chilling in reducing *Enterobacteriaceae* prevalence on pig carcasses.

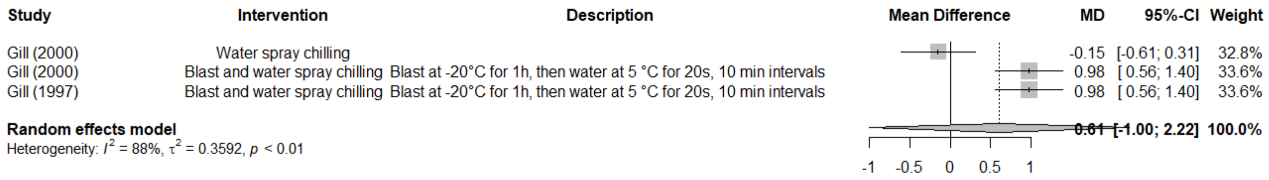


**Supplementary Figure S37**. Forest plot of the results of before-and-after trials performed under commercial abattoir conditions to investigate the efficacy of blast and water spray chilling in reducing aerobic colony count ($log_{10}$ CFU) on pig carcasses.

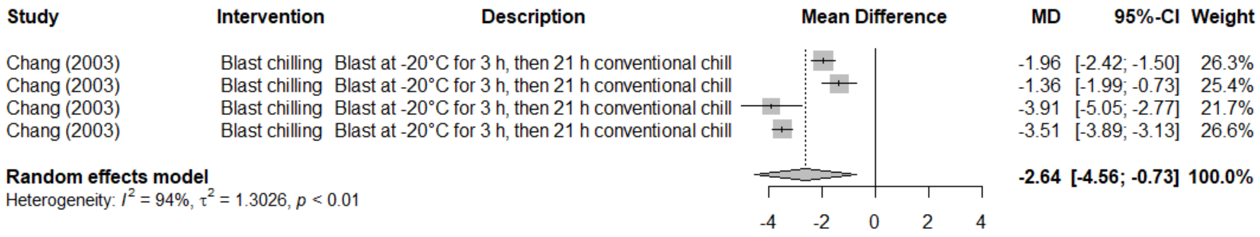


**Supplementary Figure S38**. Forest plot of the results of challenge trials performed under laboratory conditions to investigate the efficacy of blast chilling in reducing generic *E. coli* count ($log_{10}$ CFU) on pig carcass meat.

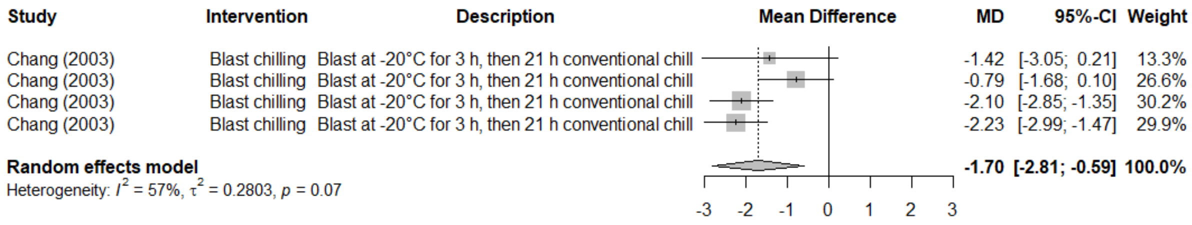


**Supplementary Figure S39.** Forest plot of the results of challenge trials performed under laboratory conditions to investigate the efficacy of blast chilling in reducing aerobic colony count ($log_{10}$ CFU) on pig carcass meat.

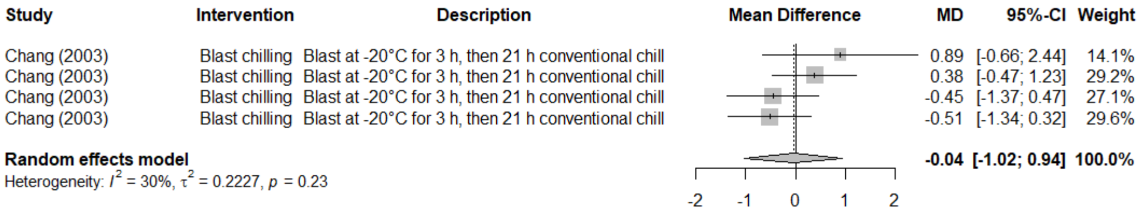


**Supplementary Figure S40**. Forest plot of the results of challenge trials performed under laboratory conditions to investigate the efficacy of blast chilling compared to conventional chilling in reducing aerobic colony count ($\log_{10}$ CFU) on pig carcass meat.

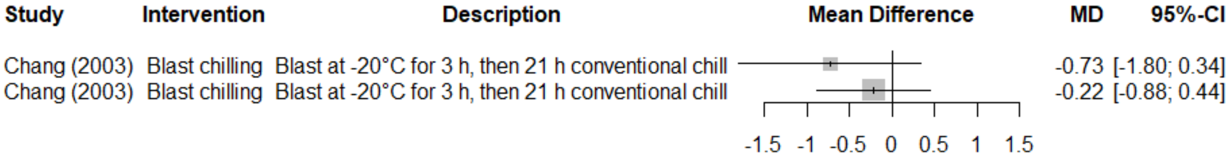


**Supplementary Figure S41**. Forest plot of the results of challenge trials performed under laboratory conditions to investigate the efficacy of blast chilling compared to conventional chilling in reducing generic *E. coli* count ($\log_{10}$ CFU) on pig carcass meat.

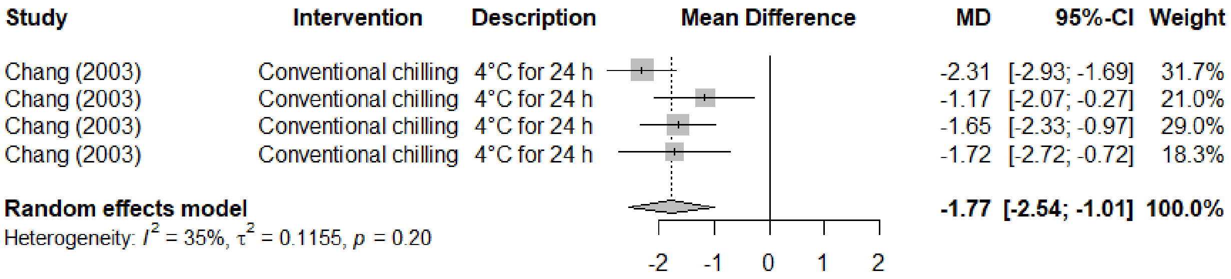


**Supplementary Figure S42**. Forest plot of the results of challenge trials performed under laboratory conditions to investigate the efficacy of conventional chilling in reducing aerobic colony count ($\log_{10}$ CFU) on pig carcass meat.

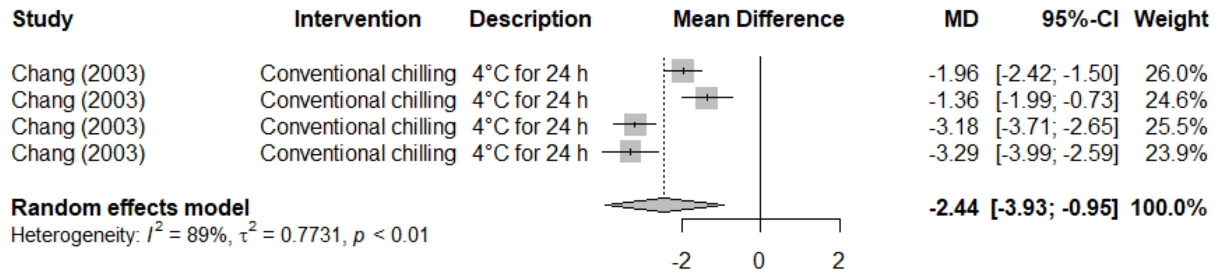


**Supplementary Figure S43**. Forest plot of the results of challenge trials performed under laboratory conditions to investigate the efficacy of conventional chilling in reducing generic *E. coli* count ($log_{10}$ CFU) on pig carcass meat.

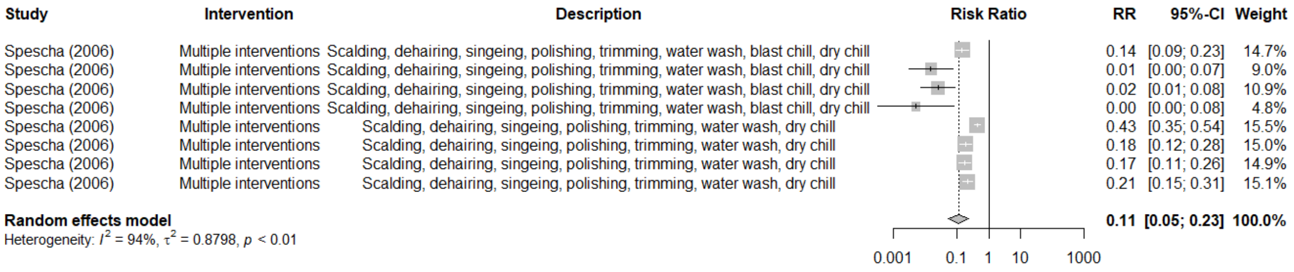


**Supplementary Figure S44.** Forest plot of the results of before-and-after trials performed under commercial abattoir conditions to investigate the efficacy of multiple interventions in reducing *Enterobacteriaceae* prevalence on pig carcasses.

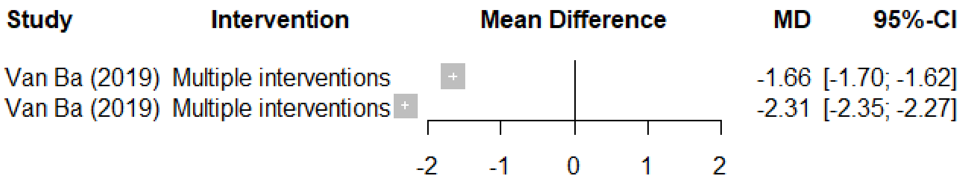


**Supplementary Figure S45**. Forest plot of the results of before-and-after trials performed under commercial abattoir conditions to investigate the efficacy of multiple interventions in reducing *E. coli* count ($log_{10}$ CFU) on pig carcasses.
